
JOURNAL INFORMATION
==============================
NLM Title Abbreviation: Ann R Coll Surg Engl
Journal ID: Ann R Coll Surg Engl
NLM Journal ID: 7506860
Journal ID: ann

Annals of The Royal College of Surgeons of England

ISSN: 0035-8843
EISSN: 1478-7083
Publisher: Royal College of Surgeons of England

ARTICLE INFORMATION
==============================
PMCID: PMC12934923
DOI: 10.1308/rcsann.2026.0028
Article ID: rcsann.2026.0028
Article version: 1
Subjects: ASiT Innovation Summit Oral Presentations

ASiT Innovation Summit Oral Presentations

Print publication date: 2026 Feb 25
Volume: 108
Issue: Suppl 1
First page: S1
Last page: S17
Copyright: Copyright © 2026, The Authors
Copyright year: 2026
License: This is an open-access article distributed under the terms of the Creative Commons Attribution 4.0 International License, which permits unrestricted use, distribution, reproduction, and adaptation in any medium, provided the original work is properly attributed.
License URL: https://creativecommons.org/licenses/by/4.0/

==============================

## Artificial intelligence in surgery

**38**

### Patient perceptions on artificial intelligence in surgery

**Aim:** Artificial intelligence (AI) is being increasingly integrated into healthcare and surgery, however there is limited data regarding patient perceptions of AI this. Understanding patient perspectives is essential to ensure responsible integration of AI. The aim of this study was to evaluate patient attitudes regarding the use of AI in surgery.
**Method:** Audit approval was obtained (CA2025-106). A questionnaire was distributed to patients ≥16 years, attending the Emergency Department in Beaumont Hospital, Dublin. Collection commenced in June 2025 and concluded in August 2025. A tally was kept of all patients approached to complete the survey. Anonymous responses were compiled and descriptive statistics were performed.
**Results:** Of 1623 people approached, 1088 responded (response rate 67%), median age group 40-49years, 50% female, and 77% White Irish. Knowledge of AI was limited, with 74% indicating little to no knowledge. 94% of respondents wished to be informed if AI was used in their care, with the majority (83%) preferring written or verbal communication. 90% of respondents favoured clinicians retaining final decision-making. With regards AI assisting with surgical planning, responses varied, 39% of respondents felt uncomfortable and 35% felt comfortable. However, the majority of patients (47%) were comfortable with AI giving advice post-operatively, with only 27% reporting they would be uncomfortable with the same.
**Conclusions:** Patients generally support AI as an adjunct to – rather than a replacement for – surgeons. Comfort is highest when AI functions alongside clinicians, and acceptance varies across clinical applications. Effective integration should prioritise transparency and accountability and seek to align patient expectations with clinical reality.

**131**

### AI driven wound assessment using image analysis

**Aim:** This systematic review aimed to evaluate the accuracy and clinical utility of artificial intelligence (AI) and machine learning (ML) in wound assessment using image analysis, focusing on burns, pressure ulcers, and surgical wounds.
**Method:** A systematic search of MEDLINE (OVID) and EMBASE was conducted for studies published from 2000 onwards. Inclusion criteria covered peer-reviewed studies evaluating AI/ML applications in wound assessment using image analysis, with expert clinical evaluation or histopathology as reference standards. Data extraction considered study design, algorithms used, input methods, output parameters, and diagnostic accuracy.
**Results:** A total of 34 studies were included (burns n = 15, pressure ulcers n = 12, surgical wounds n = 3). AI models demonstrated high performance in segmentation, depth classification, and tissue characterization, with convolutional neural networks (CNNs), U-Net, and Mask R-CNN frequently employed. Burn assessment models achieved accuracies up to 98%, significantly outperforming junior clinicians, while pressure ulcer staging models reached >90% precision and recall in several studies. Surgical wound applications were limited but showed promise in early detection of surgical site infections using thermal imaging and smartphone-based monitoring. Despite encouraging results, accuracy varied depending on wound type, imaging quality, and model sophistication.
**Conclusions:** AI and ML offer significant potential in enhancing wound assessment accuracy, efficiency, and accessibility, particularly for burns and pressure ulcers. However, evidence for surgical wounds remains limited. Wider validation on larger, standardized datasets and integration with telemedicine platforms are needed before routine clinical implementation.

**183**

### Bridging the gap in rehabilitation: real-time AI monitoring for home-based physiotherapy

**Aim:** Physiotherapy is central to recovery from musculoskeletal and neurological conditions, but lack of direct supervision often leads to poor adherence and incorrect exercise execution. To address this, we developed a deep learning-based system for real-time motion analysis, aimed at supporting remote rehabilitation with accurate feedback and monitoring.
**Method:** A custom dataset of three rehabilitation exercises (shoulder abduction, elbow flexion, standing knee raise) was collected using MediaPipe pose detection. Data underwent normalization, standardization, and augmentation before training a fully connected feedforward neural network (FNN). The model classified exercise postures as correct or incorrect, optimized with binary cross-entropy loss and Adam optimizer. For real-time deployment, webcam video streams were processed to extract pose landmarks, classify movements, and count repetitions. A Tkinter-based graphical interface enabled exercise selection, visual overlays, and progress tracking.

**Results:** The FNN achieved classification accuracy above 99% with F1-scores and precision consistently greater than 0.98 across all exercises, demonstrating robust performance and minimal overfitting. Training and validation losses converged rapidly. Real-time testing confirmed effective posture detection, automated repetition counting, and reliable visual feedback via the interface.
**Conclusions:** This study highlights the feasibility of using deep learning for accessible, real-time physiotherapy monitoring. The system enables accurate posture classification and exercise tracking, providing patients with immediate feedback to improve adherence and safety. Future work should expand exercise coverage, enhance multi-angle robustness, and validate performance in diverse real-world rehabilitation settings.

**Aim:** Cauda Equina Syndrome (CES) can cause severe neurological damage and permanent disability when left untreated. Diagnosis requires urgent MRI scans, but ~80% of referrals lack radiological evidence, causing delays in the surgical management of true positive cases. The objective of the study was to develop and validate a machine learning model for automated cauda equina compression (CEC) detection from MRI scans.
**Method:** Single mid-sagittal T2 MRI scans done for suspected CES patients from 2021-2022 were blind downloaded. Images were categorised into 4 outcome classes: normal scans, disc bulge, disc protrusion (DP), and CEC (>75% canal stenosis) scans. Saliency maps and gradient descent heatmaps were generated for those regions upon which the classification decisions were made. A 20-layer convolutional neural network with skip connections was trained on 1680 images using 5 K-fold cross validation.
**Results:** On the test set of 100 images, the model demonstrated an overall average accuracy of 97% with good discrimination between normal scans (accuracy=97%, precision=99%, recall=97%, F1 score=98%), disc bulge (accuracy=95%, precision=88%, recall=96%, F1 score=92%), DP (accuracy=97%, precision=98%, recall=97%, F1 score=98%) and CEC scans (accuracy=92%, precision=91%, recall=91%, F1 score=91%). For the individual image heatmaps, the model had high confidence in finding CEC (100%), disc protrusion (98%) and normal scans (87%).
**Conclusions:** The current study is the first of its kind to incorporate a deep learning framework in predicting the presence of CEC and as the volume of these referrals continues to grow, this tool will enable efficient diagnosis and management of CES thereby reducing patient harm and potential litigation.

**Aim:** Enhanced Recovery After Surgery (ERAS) pathways aim to improve outcomes in arthroplasty. This study sought to develop and validate machine learning (ML) models to predict discharge failure within a universal ERAS pathway and identify key patient-specific and care-specific predictive factors.
**Method:** Prospectively collected data from patients undergoing primary total hip (THA), total knee (TKA), or partial knee replacement (PKR) within a universal ERAS pathway were analysed. Supervised ML models, including Logistic Regression, Random Forest, and Gradient Boosting, were developed. SHAP (SHapley Additive exPlanations) analysis was used to determine feature importance.
**Results:** Data from 3,025 patients were analysed. ML models strongly predicted discharge failure by Day 0 or 1, with Random Forest and Gradient Boosting performing best (precision up to 0.96, recall up to 0.94, F1 up to 0.95, ROC AUC up to 0.99). SHAP analysis identified the top predictors for discharge failure: higher spinal drug concentrations (Bupivacaine > Prilocaine), higher Bupivacaine doses, no intraoperative Dexamethasone, older age, longer time to mobilisation, and joint type (TKA/THA > PKR).
**Conclusions:** Supervised ML can reliably predict discharge failure in ERAS pathways. Identifying and addressing modifiable perioperative factors, such as anaesthetic variables and rehabilitation protocols, is crucial for optimising patient outcomes and enhancing healthcare efficiency.

## Early-stage innovation

**Aim:** Traditional anatomy teaching faces challenges such as limited hands-on opportunities, difficulty visualising complex 3D structures, and variability in teaching quality due to reliance on cadavers, models, and 2D diagrams. Virtual Reality in Medicine and Surgery (VRiMS), a national organisation, seeks to address these issues through immersive VR experiences that complement conventional methods. This study assessed the educational impact of VR in anatomy education and explored its potential advantages.
**Method:** Two anatomy workshops were delivered in Bristol and Cardiff, focusing on musculoskeletal anatomy of the limbs and

ENT anatomy. Each comprised 30 minutes of didactic teaching by a consultant surgeon or anatomy lecturer, followed by 30 minutes of independent exploration using VR headsets. Participants interacted with 3D anatomical models to examine nerves, vessels, and muscle layers. Pre- and post-workshop questionnaires assessed knowledge and confidence using Likert scales and a 10-question best-of-five multiple-choice test.
**Results:** Data from 36 participants showed only 22% had prior experience with VR for anatomy learning. Pre- and post-session scores and confidence were compared using Mann-Whitney U tests. In Session 1, test scores improved from 6.42/10 to 8.08/10 (+26.0%, $p = 0.004$) and confidence from 2.50/5 to 4.50/5 (+80.0%, $p < 0.0001$). In Session 2, test scores increased from 6.17/10 to 7.00/10 (+13.5%, $p = 0.048$) and confidence from 2.46/5 to 3.96/5 (+61.0%, $p < 0.0001$).
**Conclusions:** VR-based workshops significantly enhanced anatomy knowledge and confidence. These findings highlight VR's potential as a powerful adjunct to traditional methods and suggest it could transform the delivery of anatomy education.

**113**

## Virtual reality teaching of the radial forearm flap: demonstrating the value of short, immersive sessions in overcrowded surgical curricula

**Aim:** Undergraduate exposure to complex reconstructive procedures is limited within crowded medical curricula, leaving students with few opportunities to engage with advanced surgical techniques. Virtual reality (VR) provides an immersive, accessible platform to teach anatomy and procedural steps outside of the clinical environment. Even brief exposure to VR may strengthen knowledge, confidence, and enthusiasm for surgery. This study evaluated delegate perceptions of a VR-based workshop delivered during a national surgical conference.
**Method:** The UK Plastics Research Collaborative (UKPRC) hosted *Shaping the Future of Surgery, Education, Research, and Surgical Wellbeing* on 21 June 2025. The programme included a 90-minute VR session alongside lectures, career-building workshops, and poster presentations. An online feedback form collected qualitative and quantitative data on delegates' conference experience and engagement levels, knowledge and confidence gain, and views on VR in education. Statistical significance was set at $p < 0.05$.
**Results:** Of 50 registrants, 27 completed feedback. Responders included 19 medical students, 4 clinical fellows, 3 foundation doctors, and 1 surgical trainee. Overall, 96% agreed VR effectively highlighted anatomical landmarks. The VR anatomy workshop received a mean rating of 4/5 for interest, realism, usefulness, and likelihood of reuse. Additionally, 96% supported incorporating VR into undergraduate education, and 81% reported greater confidence in understanding radial forearm free flaps after the VR session.
**Conclusions:** A single VR session significantly enhanced knowledge and confidence in a complex reconstructive technique. These findings demonstrate VR's value as a scalable, high-impact adjunct to traditional teaching, with the potential to transform surgical training if implemented across undergraduate and postgraduate curricula.

**282**

## Escape the Operating Room: A Gamified Experience for Surgical Decision-Making

**Aim:** This study explores the use of a surgically themed educational escape room to enhance medical student engagement, consolidate learning, and evaluate preparedness for clinical placements.
**Method:** A surgical escape room was developed and piloted at St Peter's Hospital for penultimate-year medical students from St George's University. Learning objectives were explicitly aligned with the GMC outcomes and mapped to the surgical undergraduate curriculum.

The escape room comprised a sequence of interlinked tasks embedded within a narrative arc. Activities included: structured A-E assessment with Sepsis 6 initiation, differential diagnosis-matching using a Jenga-style puzzle, surgical incision recognition, suturing techniques, CT interpretation, nasogastric tube insertion, and SBAR handover simulation. In April 2025, nine sessions were conducted and video recorded for ethnographic analysis. Informed consent was obtained from all participants. Following the session, participants completed structured Likert-scale questionnaires and open-ended feedback forms. Thematic analysis was applied to qualitative responses and observational data.
**Results:** All 26 penultimate-year medical students reported highly positive feedback (Likert 4.4–5.0). Participants agreed the escape room enhanced clinical reasoning, communication, and teamwork. Thematic analysis highlighted engagement, realism, and decision-making under pressure. No negative feedback emerged; several recommended longer sessions to maximise learning benefits.
**Conclusions:** Educational escape rooms represent a promising pedagogical approach in undergraduate surgical education. Their immersive and interactive nature promotes deep engagement, contextual learning, and reinforcement of essential non-technical skills. This study supports the inclusion of escape-room-based simulations as a complementary tool to traditional clinical teaching, with potential applications in both formative assessment and curriculum delivery to better prepare students for real-world surgical practice.

**320**

## AI-driven aphasia-friendly communication tool to enhance perioperative consent and patient understanding

**Aim:** Aphasia affects approximately one-third of stroke survivors, significantly impairing their ability to process written and verbal information. This creates barriers in surgical pathways where informed consent and patient-clinician communication are essential. Current solutions, such as static communication boards, are limited and lack adaptability. We aimed to develop an artificial intelligence (AI) tool that automatically converts standard medical text into an aphasia-friendly format to improve accessibility and patient understanding.
**Method:** We designed a prototype AI program that reformats conventional text into simplified language, guided by the Accessible Information Guidelines published by Stroke UK. To enhance comprehension, the tool integrates pictorial support by sourcing images from the Participics library (aphasia.ca/participics). The system was developed to generate aphasia-accessible documents such as patient information leaflets, perioperative instructions, and consent forms.

**Results:** The prototype tool successfully converts medical text into aphasia-friendly versions with simplified language and contextual images. Text transformation occurs within 30 seconds, offering a time-efficient and standardised approach compared to manually producing easy-read documents. Although formal usability testing has not yet been conducted, the tool demonstrates proof-of-concept and feasibility for clinical application.

**Conclusion:** This AI-driven aphasia communication tool represents an early-stage innovation with the potential to improve perioperative communication and informed consent in patients with aphasia. A functional online prototype has already been developed, underscoring feasibility and readiness for future validation. Next steps will focus on clinician and patient testing, integration into surgical pathways, and evaluation of its impact on patient safety and autonomy.

## Clinical research

**247**

### Multimodal analysis of surgical trainees: exploring correlations between traditional and modern assessment

**Aim:** Multiple factors influence surgical performance, including technical skills and the management of cognitive processes. Current surgical training focuses on subjective external viewership as the predominant source of feedback, but the correlation between this and modern metrics of performance are poorly understood. This study aimed to explore multimodal assessment of trainees and analyse correlations between modern and traditional methods of assessment.

**Method:** Surgical doctors were asked to perform simulated tasks on a standardised laparoscopic box trainer. Data were collected in three domains. Subjective metrics of performance were collected using validated questionnaires. Objective metrics of performance were collected using peripherally-placed wearable sabermetric physiological hardware, measuring electrodermal activity (EDA) (sweating) and heart-rate variability. Traditional measures of surgical performance were obtained by sending video recordings to Consultant Laparoscopic Surgeons, who graded using a validated scoring system. Data were analysed using a correlation matrix.

**Results:** 100% ($n = 29$) of candidates completed questionnaires and had their EDA and video recordings analysed. Heart-rate variability was difficult to obtain using the peripherally-placed hardware and was abandoned. Distractions were negatively correlated with almost all assessed aspects of surgical performance, including cognitive load (rho=0.4, $p = 0.03$), tissue handling (rho=0.41, $p = 0.029$), and overall technical performance (rho=0.41, $p = 0.029$). Poor tissue handling was correlated with situational-stress (anxiousness) (rho=0.37, $p = 0.049$), temporal demands (time-pressure) (rho=0.4, $p = 0.034$) and increased EDA (rho=0.37, $p = 0.048$).

**Conclusions:** This study proves multimodal assessment of surgical performance using technology is possible. Correlations were found between traditional and modern methods. Distractions, time-pressure and anxiousness lead to higher rates of EDA and cognitive load and ultimately affect performance.

**353**

### Predictive validity of the ACS NSQIP surgical risk calculator for postoperative complications and length of stay after lung volume reduction procedures: a single-institution study

**Aim:** The American College of Surgeons (ACS) National Surgical Quality Improvement Program (NSQIP) is a tool to help predict postoperative outcomes across multiple procedure types. Its utility across overall, serious and multiple individual complications and length of stay (LOS) for patients undergoing lung volume reduction surgery (LVRS) and endobronchial valve (EBV) procedures remains unclear.

**Method:** Data was collected retrospectively for patients operated from April 2018 to May 2025. ACS NSQIP predictions were evaluated both adjusted and unadjusted according to patients' Glenfield BFG risk profile.

**Results:** 268 patients - 151 (56.3%) male, age $64.56 \pm 8.97$ years - were included, with 178 (66.1%) in the LVRS wedge cohort and 90 (33.6%) EBV.

NSQIP showed significant predictive accuracy for serious complications (adjusted AuROC 0.630, $p = 0.008$; non-adjusted 0.619, $p = 0.014$), whereas non-significant for any complications (adjusted AuROC 0.540, $p = 0.340$; non-adjusted 0.544, $p = 0.270$).

For individual complications, NSQIP demonstrated modest discriminatory ability for pneumonia (adjusted AuROC 0.647, $p = 0.015$; non-adjusted 0.648, $p = 0.011$). However, performance was poor for surgical site infection, cardiac complications and urinary tract infection with non-significant AuROCs <0.40. No cases of venous thromboembolism, sepsis or renal failure were observed in our cohort. The correlation between NSQIP-predicted and observed LOS was Spearman $\rho$=0.453 adjusted and $\rho$=0.544 non-adjusted, both $p < 0.001$, indicating a moderately strong positive association.

**Conclusions:** NSQIP demonstrates moderate predictive validity for LOS and performs best for serious complications, but has limited accuracy for individual and overall postoperative complications. While useful for broad risk stratification, procedure-specific models may be required to optimise predictive accuracy in this subspecialist and high-risk population.

### 359

## Endoscopic flexor hallucis longus transfer with interference screw and additional tension slide cortical button for chronic achilles tendon rupture

**Aim:** Endoscopic flexor hallucis longus (FHL) tendon transfer is used in managing acute and chronic Achilles tendon rupture (ATR), including in elite athletes. A cadaveric study showed increased ultimate load with FHL tendon transfer using an interference screw and cortical button via a tension slide technique compared with screw alone. This study explored patient-reported outcomes following this modified operation.
**Method:** We reviewed imaging, history, patient-reported outcome measures (PROMs), and complications of 17 patients undergoing endoscopic FHL tendon transfer for chronic ATR using this technique. Primary outcomes were the Manchester-Oxford Foot Questionnaire (MOxFQ), EuroQol–5 Dimensions (EQ-5D), and visual analogue score for pain (VAS-Pain), with mean follow-up of 1.5 years.
**Results:** Seventeen patients (11 male, 6 female) underwent surgery between September 2020 and May 2023. Mean (SD) age was 58.3 (16.1) years and BMI 27.6 (4.8). A defined rupture event was reported in 13/17 patients (76.5%). Median (IQR) time from injury to surgery was 33 weeks (21–42). Sixteen were primary chronic ATR reconstructions, and one was revision following failed open acute repair. Postoperative MOxFQ, EQ-5D, and VAS-pain scores all improved significantly at minimum 10 months compared to preoperative values. One patient developed symptomatic tibial neuritis (5.9%).
**Conclusions:** Endoscopic FHL tendon transfer for chronic ATR, augmented with cortical button and interference screw fixation, appears safe and effective. Patients reported significant improvements in health-related quality of life, pain, and foot and ankle function.

### 361

## Preliminary radiographic classification of first metatarsal osteotomy healing following minimally invasive hallux valgus surgery

**Aim:** Minimally invasive or percutaneous surgery (MIS) for hallux valgus correction has gained popularity due to increasing evidence of favorable clinical and radiographic outcomes. However, no standardized radiographic classification exists to assess first metatarsal osteotomy healing after MIS hallux valgus surgery. This study aimed to develop a new radiographic classification system for assessing bone healing following MIS distal transverse osteotomy.
**Method:** A 4-domain radiographic system based on callus formation, anteroposterior (AP) osteotomy line, lateral osteotomy line, and remodeling for MIS osteotomy healing was developed and tested on 27 feet undergoing percutaneous transverse osteotomy for hallux valgus. Patients underwent simultaneous postoperative weightbearing computed tomography (WBCT) and standard radiographs. Five surgeons independently reviewed anonymized radiographs to assess interobserver reliability. WBCT confirmed union status and classification interpretation.
**Results:** The system demonstrated substantial interobserver reliability for lateral osteotomy line (Fleiss $\kappa=0.671$, 95% CI 0.505–0.814) and AP osteotomy line assessment ($\kappa=0.664$, 95% CI 0.459–0.811), with moderate agreement for callus formation ($\kappa=0.465$) and remodeling ($\kappa=0.439$). Classification correlated strongly with WBCT findings. An 8-point threshold differentiated union from nonunion with 85.2% overall classification accuracy. This finding was supported by the area under the ROC curve of 0.832. At this threshold, sensitivity for detecting union was 90.0% and specificity 71.4%.
**Conclusions:** This preliminary classification provides a reliable method for assessing first metatarsal bone healing after MIS hallux valgus osteotomy, with substantial interobserver reliability. It offers a standardized approach for radiographic evaluation, improving study comparability and serving as a research tool pending further validation. Its clinical applicability remains to be defined.

### 444

## Long-term outcomes of anastomotic urethroplasty for PFUI in a resource-limited setting

**Aim:** Pelvic fracture urethral injury (PFUI) is a complex condition associated with significant morbidity. Anastomotic urethroplasty (AU) is considered the gold standard for definitive management, but long-term outcomes in large series from low- and middle-income countries are rarely reported. This study evaluated functional and patient-reported outcomes of AU for PFUI over a 10-year period.
**Method:** A retrospective review was conducted of all patients who underwent AU for PFUI between January 2013 and December 2023 at a tertiary centre. Demographic, clinical, and operative details were recorded. Outcomes were assessed using uroflowmetry, post-void residual urine, erectile function scores, fertility history, and patient-reported satisfaction questionnaires. Success was defined as adequate flow rate (>15 ml/s), low residual volume (<50 ml), and no need for further intervention beyond diagnostic cystoscopy.
**Results:** A total of 440 patients were included (mean age 28.7 ± 12.5 years). Success was achieved in 79.9% (n = 350). Stricture recurrence occurred in 20.1% (n = 88), all within 6 months of surgery. Spontaneous return of erectile function was reported in

60.2% of married men, while 7.8% achieved fertility following surgery. Patient-reported outcomes indicated high satisfaction with surgical results, quality of life, and genital appearance, though erectile function and sexual performance showed variable recovery.

**Conclusions:** Anastomotic urethroplasty remains an effective treatment for PFUI, with long-term success in approximately 80% of cases. Despite the technical challenges and limited resources in low-income settings, good urinary function, recovery of erectile function, and fertility potential can be achieved alongside high patient satisfaction.

## QI and audit

**271**

### Reconstruction versus below-knee amputation for complex foot trauma: a retrospective cohort study

Katherine Poon[1], Ahmad Zargar[1], Chiraag Karia[2], Martin Taylor[2], Paul Harwood[2], Peyman Bakhsheyesh[2], Patrick Fostee[2], Rebecca Rollet[2], Waseem Bhat[2], Christopher West[2], David A Leonard[2]
[1]University of Leeds, Leeds, UK
[2]Leeds Teaching Hospitals NHS Trust, Leeds, UK

**Aim:** Complex foot trauma poses a significant management challenge, including the decision of whether to amputate or reconstruct. Evidence on outcomes is limited and inconsistent, especially within reconstruction subtypes and psychiatric burden. This study aims to compare outcomes between primary reconstruction (PR), primary amputation (PA) and secondary amputation (SA).

**Method:** A single-centre, retrospective, observational cohort study of patients managed for complex open foot fractures was conducted between January 2019 and December 2023 at Leeds Teaching Hospitals NHS Trust, UK. Variables included patient demographics, injury characteristics, operative details, time to full weight-bearing, psychiatric morbidity and healthcare utilisation. Statistical analysis was used to compare variables and outcomes, summarised as medians and interquartile ranges where appropriate.

**Results:** Fifty-eight patients were included (PR = 49, PA = 5, SA = 4). The secondary amputation cohort had a significantly greater healthcare burden than the primary amputation and primary reconstruction. Those who had free flap closure had longer hospital stay than direct closure, greater duration of operations than direct closure and SSG and greater cost of care. A quarter of patients (15/58) acquired psychiatric morbidity and were disproportionately younger than those who did not acquire psychiatric morbidity. A relationship was found between Injury Severity Score and the number of new psychiatric diagnoses.

**Conclusions:** SA was associated with the greatest clinical and economic burden, reflecting the compounded costs and morbidity of failed salvage. Younger patients were disproportionately affected by psychiatric morbidity, highlighting the need for integrated, early psychological support alongside multidisciplinary surgical care.

**299**

### Impact of undergraduate surgical conferences on medical student engagement, surgical skills confidence and career aspirations: a two-year service evaluation project

**Aim:** Undergraduate surgical conferences aim to address gaps in surgical exposure within medical curricula and foster interest in surgery. We evaluated engagement, surgical skills confidence and career aspirations across two national hybrid student-led conferences delivered in 2023 and 2024.

**Method:** Pre- and post-conference questionnaires captured demographics, surgical exposure, skills confidence and career aspirations. Data included binary, multinomial and 5-point Likert responses. Mann-Whitney U tested Likert data and unpaired t-tests multinomial comparisons.

**Results:** A total of 186 delegates attended in 2023 and 161 in 2024, with in-person attendance rising from 135 to 156. Delegates were predominantly based in Northwest England, with a near-equal gender distribution. Online participation decreased from 27.4% in 2023 to 3.1% in 2024, reflecting demand for in-person events.

In 2023, only 27.1% reported adequate curricular exposure. Surgical skills confidence improved significantly ($z = -6.84$, $p < 0.00001$), with high/moderate confidence rising from 17.8% (28/157) to 61.5% (83/135); the median shifted from 2 (minimally confident) to 4 (moderately confident). Career intentions strengthened, although not significantly, with those moderately/highly likely to pursue surgery increasing from 85.3% (185/217) to 89.8% (167/186).

In 2024, 24.7% reported adequate curricular exposure. Confidence again improved significantly ($z = 7.62$, $p < 0.00001$), with high/moderate confidence rising from 30.3% (44/145) to 73.7% (115/156); the median shifted from 2 (minimally confident) to 3 (neutral).

**Conclusions:** Undergraduate surgical conferences with practical workshops significantly enhance surgical confidence and reinforce career aspirations. Hybrid delivery ensures accessibility while supplementing limited curricular exposure. This evaluation highlights the potential of structured, student-led conferences to bridge gaps and inspire future surgeons.

**302**

### Are out-of-hours transfers for MRI in suspected cauda equina syndrome justified?

Mohammed Lester[1], Sophie Covington[2], Zakee Abdi[3], Arif Jalal[4], Ahmed K. Toma[1], Parag Sayal[1], Georgios Prezerakos[1], Anand S. Pandit[1]
[1]Victor Horsley Department of Neurosurgery, National Hospital for Neurology and Neurosurgery, London, UK
[2]Division of Medicine, University College London, London, UK

**Aim:** Cauda equina syndrome (CES) is a neurological emergency requiring timely surgical decompression within 24 hours of MRI confirmation. Limited out-of-hours (OOH) MRI access often necessitates emergency patient transfers to acute spinal centres. This study assesses the justification for such transfers.

**Method:** This retrospective cohort study analysis from April 2019 to March 2025, utilised electronic health records and referral data to UCLH (NHNN) which totalled 5525 OOH referrals for suspected CES.

**Results:** 706 patients were accepted for transfer. 285 were referred with local MRIs before referral, leading to 135 surgeries for CES. 116 were transferred after awaiting a local MRI, leading to 61 surgeries for CES. 305 patients were transferred for an urgent MRI with 37 requiring surgeries for CES. There was a significant difference in time from MRI to surgery for patients who awaited a local MRI (21.74 ± 1.67hrs) versus those transferred for an MRI (9.25 ± 2.11hrs) although both groups met the guideline target of undergoing surgery within 24 hours of a scan (MWUT=309.0, $p = 2.76e\text{-}9$).

OOH MRI sensitivity was 9.8%. Patients with a negative scan result remained hospitalised for a mean of 69.76 ± 8.37 hours until discharge, with an average admission cost of £3014.55. OOH referrals predominantly originated from doctors below ST4, with diagnostic accuracy of 4.2% compared to 5.2% for those above ST4 ($\chi^2$=0.031, $p = 0.86$).

**Conclusion:** OOH MRI transfers yield limited surgical benefit, accrue significant costs, and align poorly with GIRFT guidelines advocating for local MRIs to reduce unnecessary referrals. These findings support minimising OOH transfers for suspected CES.

**311**

## Cutting costs and boosting efficiency: reducing routine group & save testing in flexible ureterorenoscopy for better resource use and patient care

**Aim:** Flexible ureterorenoscopy (FURS) is a safe and effective procedure for renal stone management. While transient postoperative bleeding occurs in a minority of cases, transfusion rates remain minimal. This study evaluates the rate of preoperative Group and Save (G&S) testing in FURS patients before and after a review of our standard operating procedure (SOP), aiming to reduce unnecessary testing and optimise resources.

**Method:** Data were collected from theatre patient lists, including demographics, surgical indications, preoperative and postoperative haemoglobin levels, estimated blood loss (EBL), G&S ordering patterns, and postoperative outcomes. A retrospective review was conducted pre-intervention (01/10/24–31/12/24) and post-intervention (01/04/25–09/06/25).

**Results:** Prior to intervention, 51.8% (30/58) of patients had preoperative G&S tests ordered. The 30-day readmission rate was 6.9%, all related to stent issues. Following the intervention, G&S testing was significantly reduced to 20% (14/70). The 30-day readmission rate was also improved to 4.3%, also all related to stent issues. In both cycles, the mean EBL was <50 mL, and no patient required a postoperative transfusion.

**Conclusions:** Routine G&S testing for FURS is not evidence-based, and our findings suggest it is unnecessary for standard cases. Updating our SOP and providing targeted education resulted in a 31.8% reduction in unnecessary G&S testing. According to the Royal College of Surgeons, G&S tests cost £20 and require 2 hours of lab processing. This change leads to an annual saving of £1,800 and frees up 180 lab hours, improving efficiency and patient care without compromising safety.

**429**

## When the bone flap hits the floor: developing the first SOP for dropped bone flaps in neurosurgery at Oxford

Shayndhan Sivanathan, Alexandros Boukas
Oxford University Hospitals, Oxford, UK

**Background:** Dropped bone flaps during craniotomy are rare but clinically significant events that carry risks of infection, delayed reconstruction, and patient distress. The existing literature is sparse, with varied decontamination strategies and no consensus guidelines. Oxford Neurosurgery identified eight dropped bone flap incidents between March 2020 and July 2025, underscoring the absence of a standardised approach.

**Aim:** To audit local cases of dropped bone flaps, develop a microbiology-approved Standard Operating Procedure (SOP), and support safe, consistent multidisciplinary practice with improved patient transparency.

**Method:** An internal audit was conducted of all dropped bone flap incidents at John Radcliffe Hospital. Data included surgical context, management, outcomes, and governance actions. Findings were discussed at consultant morbidity and mortality meetings. In parallel, a systematic review was performed, and then microbiology and neurosurgery teams were engaged to co-develop a Trust-wide SOP. A patient information leaflet was also produced to enhance consent and autonomy.

**Results:** Eight incidents were identified. Most occurred during flap handling or fixation, with management varying from discarding bone and scheduling elective cranioplasty to immediate reconstruction with titanium mesh or PMMA. No postoperative infections occurred. Key learning points included preventive handling measures, newly introduced routine inclusion of this risk in consent, and consistent Duty of Candour. The SOP established a protocolled pathway for escalation, decontamination, and reconstruction decisions.

**Conclusion:** Dropped bone flaps are under-reported yet impactful. Through audit, multidisciplinary collaboration, and literature synthesis, we created the first SOP at our centre, standardising responses, strengthening patient safety, and providing a reproducible framework for other neurosurgical units.

**443**

## Improving inpatient administration of intravenous zoledronic acid following hip fracture: a closed-loop audit.

Zarah Wilson, Muhammad Umer Rasool, Muhammad Anas Ghazi, Mahwish Naureen
Manchester University NHS Foundation Trust, Manchester, UK

**Aim:** Hip fractures are common in older adults and carry high mortality, with one-third of patients dying within a year. Secondary fracture prevention is therefore essential. Intravenous zoledronic acid (IV Zol) reduces refracture risk by around one-third and is recommended as first-line therapy, yet many patients do not receive it due to delayed vitamin D optimisation and lack of standardised pathways. This project evaluated whether a rapid vitamin D loading policy could improve inpatient IV Zol delivery.

**Method:** Cycle 1 retrospectively reviewed patients >60 years undergoing operative femoral fracture management (2020–2022, n = 30). Standards were: (1) 100% of eligible patients should be recommended IV Zol before discharge, and (2) ≥ 90% should receive it within six months. Barriers to compliance were documented. A new policy was then introduced: high-dose vitamin D (≥160,000 IU), a pre-infusion checklist, and inpatient IV Zol administration on postoperative days 5–8. Cycle 2 (2024) prospectively assessed compliance.

**Results:** In cycle 1, all patients were recommended IV Zol, but only 17% received it within six months. Barriers included death (40%), vitamin D delays (30%), scheduling issues (23%), and clinical instability (7%). Following policy introduction, cycle 2 achieved 100% compliance, with all eligible patients receiving vitamin D loading and inpatient IV Zol on schedule.

**Conclusions:** A rapid vitamin D loading policy transformed compliance from 17% to 100%, aligning practice with national standards. This simple, sustainable intervention has the potential to reduce refracture risk, improve outcomes, and strengthen patient safety in hip fracture care.

## Collaborative research

**26**

### Neurosurgeons' leadership styles and their association with team performance and patient outcomes: a national multi-modal analysis

**Aim:** Non-technical skills are critical in high-stakes surgery, yet the leadership styles of neurosurgeons and their impact on team performance and patient outcomes remain underexplored. This study aimed to characterize neurosurgeon leadership styles and assess their association with intraoperative team behaviours and postoperative safety outcomes.

**Method:** This prospective study was conducted across seven hospitals nationally (December 2023–December 2024). Attending neurosurgeons were assessed by operating theatre (OT) team members using a validated Persian Multifactor Leadership Questionnaire (MLQ). Intraoperative team performance was evaluated through direct observation with the Observational Teamwork Assessment for Surgery (OTAS). Thirty-day postoperative outcomes were obtained from institutional databases using American College of Surgeons National Surgical Quality Improvement Program (ACS NSQIP) metrics. Associations between leadership style and outcomes were analysed with multivariable regression, adjusting for case complexity and patient risk factors.

**Results:** Eighteen neurosurgeons and 154 OT team members participated, spanning 412 surgical cases. Transformational leadership was most common (mean 3.85 ± 0.82) and was strongly associated with better team coordination (β = 0.68, p < 0.001) and communication (β = 0.72, p < 0.001). After adjustment, each 1-point increase in transformational score predicted a 22% reduction in major complications (OR = 0.78, 95% CI 0.65–0.94, p = 0.01) and a 28% reduction in surgical site infections (OR = 0.72, 95% CI 0.55–0.95, p = 0.02). Transactional leadership showed weaker, non-significant links to outcomes.

**Conclusion:** Transformational leadership in neurosurgery independently predicts better OT team performance and patient safety. Embedding structured leadership training into neurosurgical education could improve outcomes and reinforce physician-led healthcare systems.

**339**

### PECHA study: percutaneous cholecystostomy in acute cholecystitis, a multicentre cohort study

Georgios Karagiannidis[1], Dardan Uka[2], Maria Iacob[3], Roland Labinoti[3], Yousef Salem[2], Mehmet Mehmet Ergisi[4], Sophie Hall[2], Martin Sinclair[2]
[1]Imperial College London, London, UK
[2]ESNEFT, Ipswich, UK
[3]Ipswich Hospital NHS Trust, Ipswich, UK
[4]NNUH, Norwich, UK

**Aim:** To evaluate current management patterns for cholecystostomy and identify predictors of post-procedural complications.

**Method:** A retrospective review was conducted of all patients who underwent percutaneous cholecystostomy for acute cholecystitis at two UK district general hospitals between 2019 and 2023. Data collected included indication for cholecystostomy, antibiotic duration, drain duration, operator grade and post-procedure complications. Associations between key metrics and outcomes were analysed using chi-square and Fisher's exact tests (significance p < 0.05).

**Results:** Eighty-eight patients were included (median age 73, range 30–97). The most common indication for cholecystostomy was as a bridge to cholecystectomy. The median duration of antibiotics prior to intervention was 3 days (range 0–24) and the median drain duration was 28 days (range 7–131). Complications following the procedure were infrequent and included drain migration (7 cases), collection/bile leak (2), bleeding (1), fistula (1), and other specified complications (8). Operator grade was significantly associated with both post-operative (p < 0.0001) and post-cholecystostomy complications (p < 0.0001). No statistically significant associations were found between complication rates and antibiotic duration, drain duration, or patient age.

**Conclusions:** In this cohort, operator grade was the only factor significantly associated with post-procedure complications. No significant relationships were observed between complications and antibiotic duration, drain duration or age highlighting the need for further research into optimal management strategies for cholecystostomy.

**342**

### Influence of referral pathways and consultant subspecialty on morbidity and mortality in acute severe pancreatitis: a multicentre observational study

Georgios Karagiannidis[1], Ahmed Lone[2], Zahran Rahman[2], Samir Pathak[3], Martin Sinclair[2]
[1]Imperial College London, London, UK
[2]ESNEFT, Ipswich, UK
[3]Leeds Teaching Hospitals, Leeds, UK

**Aim:** To evaluate the impact of referral pathways and admitting consultant subspecialty on patient outcomes in acute severe pancreatitis (ASP).
**Method:** We conducted a retrospective analysis of 75 patients admitted with ASP across 4 district general hospitals from January 2020 to December 2024. Data were collected on demographics, referral pathways, complications and consultant subspecialty. Chi-square tests assessed associations between categorical variables. Multivariable logistic regression models were used to adjust for confounders including age, ASA score, frailty, ITU admission and early complications.
**Results:** Morbidity was significantly associated with the admitting consultant subspecialty ($p = 0.0108$). Patients under Lower Gastrointestinal (LGI) teams experienced the highest morbidity (18/26, 69%), compared to Upper Gastrointestinal (UGI) (19/42, 45%). Mortality, however, did not differ significantly by subspecialty ($p = 0.2343$). Age over 65 was independently associated with increased mortality ($p = 0.0179$). Idiopathic pancreatitis was the most common aetiology (40%) and was significantly associated with morbidity ($p = 0.0100$), mortality ($p = 0.0086$) and ITU admission ($p < 0.0001$). Referral to tertiary centres and subsequent acceptance were not independently associated with morbidity or mortality in either univariate or multivariable analyses.
**Conclusions:** Admitting consultant subspecialty significantly influences morbidity in patients with ASP, with highest rates seen in UGI-led admissions. While mortality was not significantly impacted by subspecialty or referral status, older age and DNAR decisions were strong predictors of death. These findings support the need for standardised ASP care protocols across subspecialties and underscore the importance of early frailty and escalation planning.

**351**

### ETHOS study: evaluation of hernia treatment and outcomes in pregnancy, a multicentre, cohort study

**Aim:** This study aimed to evaluate the surgical management and outcomes of pregnant women presenting with umbilical and ventral hernias and to identify factors associated with recurrence and readmission.
**Method:** A multicentre observational cohort study was conducted across five sites, including all pregnant women managed for umbilical and ventral hernias between 1 January 2016 and 31 December 2024. Data were collected retrospectively from electronic health records, capturing demographic details, surgical approach and clinical outcomes. The primary outcomes were hernia recurrence within one year of repair and readmission during pregnancy. Statistical analysis included Fisher's exact and Chi-square tests, with subgroup analysis of key clinical variables.
**Results:** Sixty-six women were included (median age 32 years). The majority presented in the third trimester (66.7%), and 60.6% were aged 25–35. Most underwent open hernia repair (74.2%), with mesh used in 34.8% of cases. Hernia recurrence within one year was observed in 5 patients (7.6%), while readmission during pregnancy occurred in 7 patients (10.6%). Recurrence was significantly more common after open repair compared to laparoscopic repair (10.2% versus 0%, $p = 0.015$). Readmission during pregnancy was also more frequent after open repair (12.2% versus 5.9% for laparoscopic, $p = 0.013$). There were no statistically significant associations between recurrence or readmission and age, parity, previous caesarean section or mesh use alone.
**Conclusions:** Open surgical repair and management by general surgery are associated with higher rates of hernia recurrence and readmission during pregnancy. Laparoscopic repair was not associated with any recurrences in this cohort. These findings may inform surgical planning and counselling for this patient population.

## Robotics and Digital Surgery

**258**

### Introducing robotic inguinal hernia repair: early outcomes from a UK NHS trust

Robyn Bartlett, Olivia Kent, Stuart Cowie, Liam Horgan
Northumbria Healthcare NHS Foundation Trust, North Shields, UK

**Aim:** The use of robotic-assisted techniques in benign general surgery is expanding in the UK, with robotic inguinal hernia repair (RIHR) emerging as a commonly performed intervention. However, comparative evidence between RIHR and traditional approaches—laparoscopic (LIHR) and open inguinal hernia repair (OIHR)—remains limited, particularly within UK healthcare settings.
**Method:** A retrospective cohort analysis was conducted at a single NHS trust, during which the first 100 RIHR were completed. Three matched elective cohorts were selected for comparison: RIHR ($n = 100$), LIHR ($n = 100$), and OIHR ($n = 100$). Variables assessed included patient characteristics, operative duration, intraoperative events, length of stay, and 30-day postoperative outcomes (including readmission, reoperation, recurrence, and mortality).
**Results:** The open approach was more frequently employed in older patients with higher comorbidity burdens, reflected in greater ASA scores. Bilateral hernia repairs were more commonly performed via minimally invasive techniques. Operative time varied significantly, with LIHR being the fastest and OIHR the longest, while RIHR showed a decreasing trend in duration over time, indicating a learning curve. Rates of same-day discharge were high (>90%) across all cohorts, though OIHR was associated with a higher rate of unplanned readmissions. There was no significant difference in thirty-day postoperative outcomes.
**Conclusions:** RIHR presents a safe and viable alternative to established surgical methods within the NHS day-case setting. Early outcomes are comparable across approaches, and robotic efficiency improves with increased experience. These results support the integration of RIHR into routine practice while underscoring the need for larger, prospective studies—particularly those evaluating long-term outcomes and cost-effectiveness.

**277**

### Virtually seamless: virtual reality for colorectal anastomosis training

Salma Mahmoud[1], Katherine Elcock[2], Deena Harji[2], Michael Bowers[3], Sonia Orsucci[3]

**Aim:** The European Society of Coloproctology reports that patients undergoing colorectal resection that develop an anastomotic leak are at increased risk of post-operative morbidity and mortality[1]. Surgical trainees must therefore acquire the necessary theoretical knowledge and technical skills for colorectal anastomosis. However, access to real-life training has become more limited, particularly following the COVID-19 pandemic[2]. Simulated environments, including virtual reality (VR), have shown promise in enhancing surgical training[3,4].
**Method:** We conducted a prospective cohort study involving 10 surgical trainees at Manchester Foundation Trust to evaluate a multimodal, consultant-led workshop on colorectal anastomosis. The workshop included theoretical teaching, a wet lab session, and VR simulation using an Oculus headset.

To measure outcomes, we used the Surgical Self-Confidence Questionnaire (SCQ), Students' Evaluation of Educational Quality (SEEQ), and the Virtual Reality Neuroscience Questionnaire (VRNQ). The SCQ was completed at baseline, after each session, and post-workshop. Trainees completed the SEEQ at the end of the workshop, while the VRNQ assessed the VR component.
**Results:** All trainees found the workshop stimulating and valuable for learning, with increased interest in the subject (SEEQ). Confidence in performing anastomosis improved progressively through each session (SCQ). For the VR simulation, 76% reported a high user experience, 87% found the game mechanics easy to use, and 89% experienced no adverse effects such as nausea or disorientation.
**Conclusions:** This study demonstrates that a multimodal workshop incorporating VR improves trainee understanding and confidence in performing colorectal anastomosis. Further research with larger cohorts is ongoing to strengthen the findings

**294**

## Survey of robotic thoracic surgery training in the UK and Ireland: interim analysis of a national, multi-centre survey

**Aim:** Robotic assisted thoracic surgery (RATS) has evolved to become a well-established surgical approach. Adequate exposure and structured training are required to prepare resident doctors for their consultant practice. This is an interim report of a multi-centre survey study exploring the current experiences of RATS training in the UK (UK) and Ireland.
**Method:** We are undertaking a cross-sectional, retrospective, web-based survey using Microsoft Forms. The data collection period spans June to September 2025. Eligible participants are resident doctors who have worked in a cardiothoracic department in the UK or Ireland between August 2024 and August 2025.
**Results:** The survey has received 76 eligible responses to date, with representation from 13 out of 14 training deaneries. Participants include trainees in cardiothoracic surgery from ST1-ST8 level (n = 49), as well as non-trainees at equivalent grades (n = 27). A total of 70% (n = 53) reported that RATS was being performed at their department. Among these respondents, only 15% (n = 8) had experienced a structured training programme for RATS. Forty percent (n = 21) did not have dual console access, or it was not being used for cases despite being in place. Respondents had acted as robotic bedside assistant for a median of 20 cases, and 51% (n = 27) had partly or fully performed cases themselves. Additional thematic analysis explored barriers and ways to improve RATS training.
**Conclusions:** Our interim report demonstrates a widespread need for increased exposure and structured training in RATS. Further data collection is ongoing, and the full evidence will be used guide a consensus on national standards for RATS training.

**307**

## Minimally Invasive but Donor-Centric: A Systematic Review and Meta-Analysis of Robotic Versus Open Living-Donor Hepatectomy

**Aim:** Robotic donor hepatectomy is performed progressively to minimise living-donor complications. However, evidence comparing donor outcomes between robotic and open hepatectomy is limited and heterogeneous. To address this gap, a systematic review and meta-analysis were conducted to compare donor outcomes between robotic and open hepatectomy.
**Method:** PubMed, ResearchGate, Wiley Online Library, and institutional resources identified cohort studies from the past six years directly comparing robotic and open donor hepatectomy. Studies reporting donor outcomes, including operative time, blood loss, complications, length of stay, transfusion, and mortality, were included. Medians/IQRs were converted to mean ± SD, and random-effects meta-analysis was performed. Sensitivity analyses excluded converted and abstract-only studies.
**Results:** Six cohort studies, including two abstract-only registry reports, met the inclusion criteria. The data analysis indicated that the operative time for robotic procedures was substantially longer (mean difference +127.3 min; 95% CI +84.0 to +170.6; $I^2$=85.2%). Robotic hepatectomy showed a nonsignificant reduction in blood loss (mean difference −152.8 mL; 95% CI −382.4 to +76.8; $I^2$=95.8%) and overall complications to the donor (OR 0.63; 95% CI 0.28–1.39; $I^2$=52.6%). Sensitivity analysis that reported mean ± SD confirmed prolonged operative time (+150.6 min; 95% CI 132.2–169.0). Donor mortality was inconsistently reported.
**Conclusions:** Robotic living-donor hepatectomy shows increased feasibility for donors, reduced blood loss, and decreased complication rates, but requires longer operative time. Heterogeneity, conversion of medians, and abstract-only data limit pooled certainty. Larger standardised multicentre studies are needed to provide robust donor-focused evidence.

**313**

## A systematic review and meta analysis comparing clinical and functional outcomes of Mako-assisted versus conventional total knee replacement (PROSPERO ID: CRD420251115864)

**Aim:** To systematically evaluate the clinical effectiveness of Mako total knee arthroplasty compared with conventional jig-based TKA, focusing on functional outcomes, implant positioning, complications, length of stay (LOS), postoperative pain, and recovery time.
**Method:** A systematic review and meta-analysis was conducted following PRISMA guidelines. Prospective, retrospective, and registry-based comparative studies were included. Data were extracted for validated functional scores (OKS, KOOS-JR, VR-12), alignment parameters (hip–knee–ankle angle, outlier rates), perioperative complications, LOS, and VAS pain at multiple timepoints. Random-effects models were used for quantitative synthesis; heterogeneous outcomes (time to recovery milestones, gait analysis) were synthesised narratively. Certainty of evidence was assessed using GRADE.
**Results:** Twenty-one studies (n = 51,226) were included. At 12 months, robotic patients demonstrated modest improvements in functional outcomes (OKS pooled MD +2.5, 95% CI +1.0 to +3.9), approaching the minimal clinically important difference. Robotic TKA reduced alignment outliers. There was no consistent difference in periprosthetic joint infection or early revision rates. LOS was ∼0.5 days shorter with robotic, an effect confined to non-ERAS pathways. Pain outcomes showed no acute benefit, but a 6-month meta-analysis (Yang 2024, Masilamani 2025; n = 286) demonstrated lower pain with robotic (MD −0.43, 95% CI −0.57 to −0.28). Narrative synthesis indicated faster inpatient recovery milestones in conventional care but no advantage under ERAS protocols.
**Conclusions:** Robotic TKA confers small but consistent benefits in functional outcomes, alignment precision, LOS, and mid-term pain, without clear reductions in major complications. These advantages are context-dependent, most evident outside ERAS pathways. Higher-quality RCTs are required to confirm long-term clinical relevance.

**325**

### Colorectal trainees as primary console surgeons in elective robotic assisted surgery: evaluating outcomes of a robotic colorectal training programme

Muhammad Bilal Akbar, Aye Aung, Yousef Abduldaiem, Maaz Maghazi, Claire Carden, Adarsh Shah
NHS Tayside, Dundee, UK

**Aim:** Patients have benefited from the widespread rapid adoption of robotic assisted colorectal surgery (RAS). The surgical training landscape has therefore had to adapt, with upskilling of established laparoscopic colorectal surgeons and concurrent RAS training for colorectal trainees. Patient outcome data of colorectal trainees as primary console operators is not widely published. We report outcomes of the Scottish Robotic Colorectal Training Program (RCTP).
**Method:** The RCTP commenced in March 2024 following multistakeholder collaboration (NHS Tayside, NES, RCSEd, and Intuitive). Six colorectal trainees undertook the 4-phase RCTP, supervised by three established consultants (including a proctor). Outcomes for elective colorectal resections performed by trainees as primary console operators under consultant supervision were compared with those of consultant surgeons as primary operators.
**Results:** Data collected from 21/02/2024 to 26/06/2025. 68 elective resections were performed: trainees = 31, consultants = 37. These comprised segmental colonic resections, total/subtotal colectomies, and rectal resections, which trainees completed robotically to meet ARCP requirements. Mean operative times were similar for all procedures. Trainees had lower R1 resection and conversion-to-open rate but marginally higher anastomotic leak rate and length of hospital stay. No mortality noted in both groups.
**Conclusions:** Our RCPT demonstrated comparable outcomes for elective colorectal resections, especially trainee-led rectal resections, thereby encouraging existing training programmes to incorporate robotic colorectal training. This should be paralleled with development of RAS-specific workplace-based assessments and formal evaluation of patient outcomes.

## Systematic review and meta-analyses

**191**

### Comparing post-operative wound outcomes of negative pressure wound therapy (NPWT) vs standard dressings in emergency laparotomies: a systematic review and meta-analysis

**Aim:** While prior evidence has suggested better post-operative wound outcomes when using negative pressure wound therapy (NPWT) in emergency laparotomies, recent large-scale studies have reported conflicting findings, creating clinical uncertainty regarding its true efficacy. We conducted a meta-analysis to synthesize the latest evidence evaluating the efficacy of NPWTs compared to standard dressing for improving post-operative wound outcomes in patients undergoing emergency laparotomy.
**Method:** We searched PubMed, Embase and Cochrane library for studies comparing NPWT and standard wound management in emergency laparotomies. We computed odds ratios (ORs) with 95% confidence intervals (CIs) and conducted separate subgroup analyses for randomized control trials (RCTs) and observational studies. Data were pooled using a random-effects model. All statistical analyses were conducted using the R software, version 4.5.0.
**Results:** We included 7 RCTs and 9 observational studies, comprising of 71,337 patients who underwent emergency laparotomy. The use of NPWT was found to have reduced SSIs in both RCT (OR 0.34; 95% CI 0.19 to 0.64; p = 0.0006) and observational studies (OR 0.5; 95% CI 0.32 to 0.78; p = 0.0021). Hospital readmissions were also significantly reduced in observational studies (OR 0.67; 95% CI 0.46 to 0.98; p = 0.0412). No significant effect on wound dehiscence, collection formation, mortality nor length of hospital stay were found.
**Conclusions:** Our findings reinforce the established benefit of NPWT in lowering SSI rates and provide new evidence of its association with significantly reduced hospital readmission rates following emergency laparotomy, underscoring its potential to improve overall patient outcomes and resource utilization.

**201**

### The risk of recurrent anterior cruciate ligament injury in male and female football players following anterior cruciate ligament reconstruction: a systematic review and meta-analysis

**Aim:** Anterior cruciate ligament (ACL) injuries in footballers have devastating career impacts. The incidence proportion (IP) of index ACL injury in footballers is 2.7%, however, the IP of recurrent ACL injury in football is unknown. Therefore, the purpose of this meta-analysis was to determine the IP of recurrent ACL injury following unilateral ACL reconstruction (ACLR) in footballers and to examine the sex-specific differences.
**Method:** A systematic review and meta-analysis was performed. Four databases were searched. The eligibility criteria included studies reporting both the recurrent ipsilateral and contralateral ACL injuries of footballers after ACLR with at least two years post-operative follow up.
**Results:** The database search yielded 3,204 records, of which, 19 studies were included. The pooled IP of recurrent ACL injury was 21.3%. There was no significant difference in risk between ipsilateral graft and contralateral ACL injury (RR = 1.09, 95% CI: 0.84 to 1.41, $I^2 = 63\%$), with an IP estimated at 9.8% and 10.8%, respectively. There was no significant difference in risk of recurrent ACL injury between females and males (RR = 1.29, 95% CI: 0.95 to 1.74, $I^2 = 56\%$), with an IP of 23.0% and 15.7%, respectively. Although the risk of ipsilateral reinjury was similar between sexes (RR = 0.93, 95% CI: 0.48 to 1.80, $I^2 = 70\%$), females had a significantly greater risk of contralateral injury than males (95% CI: 1.09 to 2.16, $I^2 = 0\%$).
**Conclusions:** The IP of recurrent ACL injury in footballers was 21.3%. Females had a significantly increased risk of contralateral ACL injury than males, identifying an area to improve the rehabilitation programmes of female footballers.

**336**

## Autologous lymphatic vessel transplantation for extremity lymphedema: a systematic review and meta-analysis

**Background and Aim:** Extremity lymphedema is a chronic, progressive condition that impairs physical function, psychological wellbeing, and quality of life. Microsurgical techniques including lymphovenous anastomosis (LVA) and vascularised lymph node transfer (VLNT) have improved outcomes, though donor-site morbidity and variable efficacy remain concerns. Lymphatic vessel transplantation (LVT), in which functional autologous lymphatic collectors are harvested and transplanted as free grafts, has emerged as an alternative reconstructive strategy. This study provides the first systematic review and meta-analysis evaluating its clinical effectiveness and safety.
**Method:** A PRISMA-compliant systematic review and meta-analysis was performed across major databases. Eligible studies reporting clinical, functional, patient-reported, or donor-site outcomes following LVT were included. Risk of bias was assessed, and pooled analyses conducted where appropriate.
**Results:** Nine studies (one case report, seven case series, one prospective cohort), all from Germany, were included. Donor lymphatic collectors were most frequently harvested from the medial thigh. Meta-analysis demonstrated a 35% pooled incidence of donor-site complications (95% CI 20–50%). LVT achieved significant reductions in limb volume (arm: −830.18 cm³; leg: −2493 cm³) and transport index (−10.15, 95% CI 5.49–14.82), with no donor-site lymphedema. Patient-reported outcomes showed improvements in physical (arm: + 4.40; leg: + 2.21) and psychological domains (arm: + 3.85; leg: + 1.60), alongside reduced burden of conservative therapy. Comparative analyses indicated fewer and less severe donor-site complications than VLNT.
**Conclusions:** LVT is a promising microsurgical option for extremity lymphedema, offering meaningful improvements in function, quality of life, and treatment burden, with relatively low donor-site morbidity.

**414**

## Focused appendiceal CT as an innovative diagnostic pathway, a systematic review and meta-analysis

**Aim:** Computed tomography (CT) remains central to diagnosing acute appendicitis for indeterminate cases despite clinical scoring systems such as Alvarado and AIR. However, increasing reliance on CT raises concerns about radiation exposure and resource use. This study evaluated the diagnostic accuracy of Focused appendiceal CT (FACT), highlighting its potential implementation as a diagnostic innovation within future clinical pathways.
**Method:** A systematic search of Cochrane Library, PubMed, MEDLINE, EMBASE, EMCARE, and Ovid MEDLINE identified 22 eligible studies, with 15 suitable for quantitative extraction. Sensitivity, specificity, and confusion matrices were obtained alongside scanner details and reported radiation doses. Meta-analysis using a bivariate model and HSROC analysis was performed. Study quality was assessed with QUADAS-2.
**Results:** The 15 included studies varied in design and patient selection; six used non-contrast CT, nine used contrast. Overall pooled sensitivity and specificity were 94.5% and 94.6%. Contrast-enhanced FACT outperformed non-contrast (sensitivity 97.8% vs 85.8%; specificity 96.9% vs 85.8%). Two studies reported radiation estimates: one showed a reduction to 3.6 mSv from 7 mSv for standard CT; the other, 7.7 mSv from 9 mSv.
**Conclusions:** FACT demonstrates comparable accuracy to standard CT (sensitivity 94.5% vs 95%; specificity 94.6% vs 94%). Importantly, contrast enhancement of any form dramatically improved performance. With evidence suggesting radiation reduction of approximately one-third compared with standard CT. FACT represents an innovation that is both safe and effective, supporting its integration into evolving clinical pathways for the diagnosis of appendicitis.

**434**

## Surgical versus nonsurgical management of distal radius fractures: a systematic review and meta-analysis highlighting functional outcomes and age-based considerations

**Aim:** Distal radius fractures (DRFs) are among the most common upper limb injuries. Management varies between surgical fixation and conservative treatment, and the choice remains debated, particularly regarding functional recovery across age groups. This systematic review and meta-analysis evaluated comparative functional and radiological outcomes to guide evidence-based practice.
**Method:** A systematic search of Medline, Embase, Cochrane, and Google Scholar (2012–2023) was performed using predefined Boolean terms. Prospective and comparative retrospective studies reporting functional outcomes were included. Two reviewers independently extracted data. Pooled analysis was conducted using random-effects models. Bias was assessed using the Cochrane tool, and heterogeneity was evaluated with $I^2$ statistics.
**Results:** Fourteen studies including 1,310 patients were analysed. Surgical intervention significantly improved Disability of the Arm, Shoulder, and Hand (DASH) scores compared with nonsurgical management (WMD = 3.9, 95% CI: 2.0–5.9, $p < 0.001$). Younger patients (<65 years) showed greater functional benefit. Surgical treatment also improved radial inclination, radial length, volar tilt, and forearm rotation. No significant differences were found in wrist flexion, extension, or deviation. Grip strength decline was more pronounced in younger surgically treated patients compared with older counterparts.
**Conclusions:** Surgical treatment of DRFs yields superior functional and radiological outcomes in younger patients, while older individuals may achieve comparable results with non-surgical care. Age, functional expectations, and risk stratification should inform treatment decisions. These findings highlight the role of evidence synthesis in guiding clinical practice and improving patient outcomes.

## Global surgery

**23**

### Improving the timeliness of surgical antibiotic prophylaxis in cranioplasty: a three-cycle closed-loop audit at a tertiary neurosurgical center

**Aim:** Surgical site infection (SSI) following cranioplasty is a serious complication linked to morbidity, implant loss, and increased healthcare costs. Administering surgical antibiotic prophylaxis (SAP) within 60 minutes before skin incision is a global standard for SSI prevention, yet adherence is often inconsistent. We aimed to measure and improve compliance with the 60-minute SAP timing standard in cranioplasty patients.
**Method:** A prospective, three-cycle, closed-loop audit was conducted at Razavi Hospital (June 2020-March 2021). Adult patients undergoing elective or semi-urgent cranioplasty were included. Cycle 1 assessed baseline compliance. Cycle 2 introduced two interventions: mandatory scrub-side verbal confirmation during the surgical "Time Out" and a nurse-led pre-theatre reminder. Cycle 3 added an automated Electronic Health Record (EHR) prompt for the anaesthetic team. Primary outcome: percentage of cases compliant with the 60-minute window. Secondary outcome: 90-day SSI rate.
**Results:** A total of 150 patients were included across the three cycles (n = 48 in Cycle 1, n = 52 in Cycle 2, n = 50 in Cycle 3). Compliance with the 60-minute SAP window improved from a baseline of 47.9% in Cycle 1 to 84.6% in Cycle 2, and to 96.0% in Cycle 3 ($p < 0.001$). This improvement was correlated with a statistically significant reduction in the 90-day SSI rate, which decreased from 10.4% in Cycle 1 to 5.8% in Cycle 2, and to 2.0% in Cycle 3 ($p = 0.048$).
**Conclusions:** A staged quality improvement approach from human-led checks to automated EHR prompts, significantly enhanced SAP timing compliance and reduced SSIs. This audit offers a replicable model for improving patient safety in high-risk neurosurgical procedures.

**135**

### Laparoscopic versus open cholecystectomy in low- and middle-income countries: a systematic review and meta-analysis of perioperative and postoperative outcomes

**Aim:** This study aims to compare perioperative and postoperative outcomes between laparoscopic cholecystectomy (LC) and open cholecystectomy (OC) in low- and middle-income countries (LMICs).
**Method:** A systematic review and meta-analysis were conducted to evaluate studies comparing LC and OC in LMICs as defined by the World Bank classification. The primary outcomes included surgical site infection (S SI), postoperative ileus, and length of hospital stay (LOS). Secondary outcomes assessed were operation time, intra-operative bleeding, bile spillage, bile leak, and bile duct injury. Data were synthesised using a random-effects model, and results were expressed as risk ratios or mean differences with 95% confidence intervals.
**Results:** Twenty-four studies involving 4,389 patients (LC = 2,247; OC = 2,142) were eligible to be included in this review. LC was associated with a significantly reduced risk of SSI and postoperative ileus compared to OC [Odds Ratio (OR) 0.34, $P = 0.00001$] and [OR 0.42, $P = 0.04$], respectively. Additionally, LC resulted in a shorter LOS [$P = 0.00001$]. No significant differences were found between the two groups in terms of operation time [$P = 0.67$], intra-operative bleeding [$P = 1.0$], bile leak [$P = 0.17$], or bile duct injury [$P = 0.12$]. However, OC was associated with a significantly lower risk of intraoperative bile spillage [$P = 0.05$].
**Conclusions:** In LMICs, LC offers clear advantages over OC by reducing complications and expediting recovery, despite similar intra-operative safety outcomes. These findings support LC as the preferred approach in resource-constrained settings where adequate expertise and facilities are available.

**198**

### Brachial plexus injuries in Cambodia: demographics, interventions, and post-operative outcomes

**Aim:** Brachial plexus injury (BPI) is a major cause of disability in low- and middle-income countries due to road traffic accidents. There are no studies reporting the epidemiology of BPIs in Cambodia. Therefore, the purpose of this study was to report the demographics, surgical interventions, and post-operative outcomes of surgically treated BPIs at the Children's Surgical Centre (CSC), Cambodia, and reflect on the service development.
**Method:** We retrospectively reviewed all surgically treated BPIs at CSC between January 1, 2012 and April 30, 2025. Extracted data included patient demographics, injury mechanism, BPI subtype, operative details, injury to surgery interval, international involvement, and ≥12-month post-operative elbow flexion and shoulder abduction Medical Research Council (MRC) strength.
**Results:** 319 patients had 439 BPI operations at CSC. Mean age was 27.21 years (range: 2 months – 61 years) and 291 (91.20%) were males. The aetiology was blunt trauma in 296 (92.79%) and the most common subtype was pan-plexal injury in 177 (55.5%) patients. Mean injury to index operation interval was 7.52 months. Annual case volume increased up to 2019 and international surgical involvement shifted to Cambodian involvement. At ≥12 months post-operatively, 52/74 (70.27%) and 36/53 (69.92%) achieved ≥3 MRC strength in elbow flexion and shoulder abduction, respectively.
**Conclusions:** There is a high BPI burden among young males following road traffic accidents in Cambodia. CSC has developed a sustainable, self-sufficient BPI service with encouraging functional outcomes. Future priorities include earlier referral after BPI injury and improved outcome capture.

**200**

## Outcomes of collagen and conventional dressings in diabetic foot ulcers: a comparative study in Bangladesh

**Aim:** Diabetic foot Ulcers are a major consequence of diabetes and major cause of lower-extremity amputation. Effective management are essential. In Bangladesh, DFU is becoming a serious health issue. The purpose of this study was to compare the effectiveness of collagen and traditional dressings in the treatment of DFU patients.
**Method:** An observational comparative cross-sectional study was conducted in the Dept. of Surgery, Dhaka Medical College Hospital, over six months. A total of 100 patients with DFU were enrolled and equally divided into two groups: one treated with collagen dressing and other with conventional dressing. Patients were recruited following inclusion and exclusion criteria, and consent was obtained. Data on socio-demographic characteristics, glycemic control, and treatment outcomes were collected. Outcome assessment focused on time to granulation and complete healing. Data were analyzed using STATA (version 13).
**Results:** The mean age of participants was 60.07 ± 10.39 years (range: 34–78 years); 77% were male. Mean HbA1c was comparable between groups (collagen: 8.12 ± 1.14% vs. conventional: 8.05 ± 0.74%). Granulation occurred significantly earlier in the collagen groups (2.26 ± 1.58 weeks) than the conventional groups (3.76 ± 1.57 weeks; $p < 0.001$). Similarly, mean healing time was shorter with collagen (4.90 ± 2.54 weeks) compared to conventional dressing (6.24 ± 3.76 weeks; $p < 0.05$). Hazard ratio analysis showed granulation tissue appeared 1.96 times more likely with collagen dressing than conventional (HR: 1.96; 95% CI: 1.31–2.96; $p < 0.05$).
**Conclusions:** Diabetic foot ulcers heal slowly and carry a high infection risk. This study found that collagen dressings outperform conventional ones, promoting faster healing and early granulation.

**308**

## Breaking barriers: establishing robotic surgery in a developing country

Mushtaq Hussain[1,2], Rehan Mohsin[2], Harris Qureshi[2], Riaz Leghari[2], Naveed Mahar[2]
[1]Queen Elizabeth Hospital, Birmingham, UK
[2]Sindh Institute of Urology and Transplantation, Karachi, Pakistan

**Aim:** Robotic surgery offers clear clinical advantages, yet its uptake in developing countries is limited by financial, logistical, and training barriers. This study describes the outcomes of a robotic program over five years, highlighting the challenges faced and the enablers that supported its growth in a resource-limited setting.
**Method:** All robotic surgeries performed between 2017 and 2021 were retrospectively reviewed. Patient demographics, surgical procedures, and perioperative outcomes were recorded. Outcomes included intraoperative blood loss, system malfunction, conversion to open surgery, and mortality. Trends in procedural volume and case complexity were also analysed to reflect programme development.
**Results:** A total of 550 procedures were completed, with a mean patient age of 39.6 years (±16.8). The mean blood loss was 180 ml (±120). Three cases (0.5%) of system malfunction were reported, 42 cases (7.6%) required conversion to open surgery, and mortality was 1.1% (n = 6). Initially dominated by nephrectomies and pyeloplasties, the programme has since expanded to include more complex operations. The most frequent cases now are radical prostatectomy, partial nephrectomy, and radical cystectomy with intracorporeal ileal conduit, reflecting both surgeon expertise and institutional capacity growth.
**Conclusions:** Despite significant barriers, robotic surgery was successfully introduced and scaled in a developing country, with safe outcomes comparable to international benchmarks. Strong institutional support, structured training, and collaborative partnerships were critical enablers. These findings demonstrate that advanced surgical innovation can be established in low- and middle-income settings when both technical and organizational challenges are addressed.

**355**

## Systematic review of nonoperative joint-preserving strategies in sickle cell disease-related avascular necrosis: implications for low-resource healthcare systems

Emmanuel Oladeji[1,2], Oluwatobi Olayode[1,3], Abdulahi Zubair[1], Patrick Okonkwo[1], Onyeka Omerenma[1], Oghofori Obakponovwe[2]
[1]Surgery Interest Group of Africa, Lagos, Nigeria
[2]St Richard's Hospital, Chichester, UK
[3]Obafemi Awolowo University Teaching Hospital, Ile-Ife, Nigeria

**Aim:** Avascular necrosis (AVN) is a debilitating complication of sickle cell disease (SCD), with a disproportionate burden in low-resource settings where access to surgical management is often limited. However, existing reviews have focused mainly on operative interventions. This systematic review examines the efficacy and safety of nonoperative strategies, which may offer more feasible and contextually relevant options in such settings.
**Method:** A systematic search of PubMed, Embase, Cochrane Library, and regional databases was conducted up to April 2025. Eligible studies reported outcomes of nonoperative interventions in SCD patients from African, Asian, Caribbean, and Latin American settings.
**Results:** Seven studies (n = 285) involving pre-collapse AVN mainly affecting the femoral head were included. Interventions included cell-based therapies (bone marrow mononuclear cells, mesenchymal stromal cells, or cultured osteoblasts), physical therapy, and intravenous bisphosphonates. Cell-based therapies were the most common, showing the most consistent improvements in pain, function (Harris Hip Score increase >40 points), and radiological stability, with few complications. The effectiveness of physical therapy was demonstrated in a randomised trial with a mean improvement in the Children's Hospital Oakland Hip Evaluation Scale scores of 15.7 points. Meanwhile, intravenous bisphosphonates proved effective in reducing bone pain.
**Conclusions:** Cell-based therapies emerged the most effective for early SCD-related AVN, although cost and resource requirements may restrict access in low-resource settings where the disease is most prevalent. Physical therapy remains a practical, resource-efficient alternative. Intravenous bisphosphonates are a promising non-invasive medical option; however, larger, well-controlled studies are necessary to validate these findings and inform clinical decisions.

**427**

### Low level of awareness regarding prostate cancer and screening among men at risk in rural Gezira State, Central Sudan: a multi-centered cross-sectional study

**Aim:** Prostate cancer (PC) is the most common cancer affecting Sudanese men. In central Sudan, most of PC cases are rural inhabitants, present with large tumor and discovered at late stage. This study aimed to assess the awareness of men aged 40 years or more regarding prostate cancer and screening in central Sudan.
**Method:** This was a descriptive cross-sectional study included randomly selected 408 males who attended at nine rural hospitals between 22end and 27th of February 2023. Data was collected using a validated, pre-tested structured questionnaire, and analyzed by SPSS version 26.
**Results:** The mean age of participants was 53.5 years ± 10.5 years and the majority were farmers (61.8%). Nearly half of participants (44.9%) had inadequate awareness about PC. About one-third (37%) never heard about PC, while (31.6%) heard about PC screening. About (10.3%) had a history of prostate related problem. Regarding PC symptoms, participants were unable to identify difficulty starting urination and blood in urine/semen as symptoms of PC, with (30.6%) and (80.4%) respectively. A minority recognized digital rectal examination (10%) and prostate specific antigen (30%) as PC screening tests. Only (28%) of participants were aware of family history as a risk factor for PC.
**Conclusions:** The findings of this study revealed a significant gap in knowledge regarding PC and screening among men in rural Gezira state. This may delay PC discovery and treatment leading to increased mortalities. We recommend organizing a national awareness campaign to raise the awareness about PC and encourage men to seek screening services.

## Best Top 5

**184**

### Efficient and explainable machine learning for dermoscopic image classification

**Aim:** Early and accurate recognition of malignant skin lesions, particularly melanoma, is critical for surgical planning and improving survival outcomes. Current diagnostic practice relies heavily on visual inspection, which is prone to variability. While deep learning models offer high accuracy, their complexity and resource requirements restrict use in surgical clinics. This study evaluates whether tree-based ensemble methods—Random Forest (RF) and Gradient Boosting (GB)—can provide a reliable, interpretable, and clinically applicable alternative for skin lesion classification.
**Method:** A dermoscopic dataset of 8,000 images, representing basal cell carcinoma, benign keratosis-like lesions, melanocytic nevi, and melanoma, was processed using standard augmentation and feature extraction techniques (texture, color histograms, and gradient analysis). RF and GB classifiers were trained with optimised hyperparameters and benchmarked against a lightweight convolutional neural network. Model performance was assessed using accuracy, macro-F1, and area under the ROC curve (AUC). Clinical relevance was explored through feature importance mapping with Shapley Additive Explanations (SHAP).
**Results:** Gradient Boosting achieved the highest performance (accuracy 89%, macro-F1 0.88), closely followed by Random Forest (86%, 0.85). Both models exceeded an AUC of 0.94 for melanoma detection, comparable to the deep learning benchmark while training faster. SHAP analysis highlighted pigmentation irregularities and border textures as key features, reflecting established surgical diagnostic cues.
**Conclusions:** Tree-based ensembles with engineered features can rival compact deep learning models in skin lesion classification while offering advantages in speed and explainability. These findings support their potential integration into resource-limited or point-of-care diagnostic settings.

**281**

### Extent of resection in meningioma surgery: concordance and prognostic value of surgical, radiological, and volumetric assessments

Matthew Deehan[1], Dermot Mallon[2], Shalwin Mathew[1], Gillian Pace[1], Neil Kitchen[1], Harpreet Hyare[2], Parashkev Nachev[3], Hani Marcus[1,4], Anand Pandit[1]
[1]Victor Horsley Department of Neurosurgery, National Hospital for Neurology and Neurosurgery, London, UK
[2]Lysholm Department of Neuroradiology, National Hospital for Neurology and Neurosurgery, London, UK
[3]High Dimensional Neurology, Queen Square Institute of Neurology, London, UK
[4]TeQ Group, Queen Square Institute of Neurology, London, UK
Postoperative meningioma recurrence poses a significant clinical challenge. Extent of resection (EOR) is a key predictor, and the Simpson Grading (SG) scale has historically been utilised to estimate such. No consensus exists for which of SG, postoperative radiology report (RG), and residual tumour volume (RTV) best predicts recurrence.

**Aim:** (1) Evaluate the concordance between intraoperative and postoperative EOR and (2) determine which estimate has the greatest prognostic value for predicting tumour recurrence.
**Method:** In this retrospective review of 270 patients, intraoperative EOR was compared to EOR from postoperative imaging. Agreement was assessed using Cohen's Kappa and absolute agreement. Machine learning models (logistic and Cox regression) were developed to compare the prognostic performance of SG, RG, and RTV for predicting recurrence.
**Results:** Agreement between surgical and radiological EOR was substantial (κ=0.704). Machine learning models demonstrated that imaging-based metrics consistently outperformed SG. RTV performed best for 3- and 5-year prediction (AUROC = 0.787, 0.709). RTV Cox models also performed best (C-index = 0.752). No significant recurrence risk difference was found between SG 1,2, and 3.
**Conclusion:** Objective, imaging-based metrics are superior to the subjective Simpson Grade for predicting meningioma recurrence, with quantitative residual tumour volume offering the most robust prognostic value. To our knowledge, this is the first study to directly compare these EOR estimators within a machine learning framework. These findings support a shift away from a reliance on the Simpson Grade and towards the integration of quantitative radiological data for more accurate postoperative risk stratification and patient management.

**285**

## NHS oral cancer patient-facing information: enhancing readability and preserving content with large language models

**Aim:** Early diagnosis is crucial for improving oral cancer prognosis and surgical outcomes. Patient-facing information leaflets support recognition, referral, and treatment planning, but are often written above recommended reading levels. This may limit accessibility, delay presentation, and reduce patients' ability to engage in shared decision-making, including surgical options where relevant. Large language models (LLMs) may offer a solution by simplifying health information while maintaining content fidelity.
**Method:** Patient information leaflets were collected from NHS-affiliated websites, including general cancer education and treatment options. Original and LLM-simplified texts (ChatGPT, Claude, Gemini) were assessed using validated readability tools (FRES, FKGL, GFI, CLI, SMOG). Content fidelity was evaluated using character 3–5-gram cosine, sentence-content retention, and latent semantic analysis (LSA).
**Results:** LLM revisions significantly improved readability compared with original texts across all five indices ($p < 0.0001$). Original texts had a mean FRES of $66.4 \pm 7.7$, while Claude ($81.6 \pm 6.2$) was the only model to surpass the recommended benchmark. All LLM outputs demonstrated high semantic similarity to the originals (mean LSA $0.97 \pm 0.04$; $0.94 \pm 0.09$; $0.96 \pm 0.08$) with strong character 3–5-gram similarities ($0.85 \pm 0.05$; $0.80 \pm 0.08$; $0.82 \pm 0.08$).
**Conclusions:** LLM-based simplification enhanced the readability of NHS oral cancer patient information while preserving content. Improved accessibility may support earlier presentation, referral, and engagement in surgical and non-surgical treatment decisions. Claude was the only model to exceed the benchmark readability threshold. LLMs show promise for strengthening communication in surgical oncology, but optimisation and human oversight remain essential.

**447**

## Total thyroidectomy or hemithyroidectomy for low-risk differentiated thyroid carcinoma?

**Aim:** Optimal surgical management for low-risk differentiated thyroid cancer (DTC) remains controversial, particularly regarding initial hemithyroidectomy versus total thyroidectomy. Understanding factors predicting the need for completion thyroidectomy and residual disease in the remnant lobe is critical for risk-adapted treatment.
**Method:** We conducted a multicentre retrospective cohort study of 387 patients undergoing surgical management of DTC between 2015 and 2024 across three tertiary centres. Demographic, clinical, cytological, and pathological data were extracted and compared by surgical extent. Among patients initially treated with hemithyroidectomy, predictors of proceeding to completion thyroidectomy were assessed. In patients undergoing completion thyroidectomy, clinicopathological factors associated with residual disease in the completion lobe were identified using both univariate and multivariate logistic regression analyses.
**Results:** Of 387 patients (median age 44 years, 78% female), 243 (63.0%) initially underwent hem, and 143 (37.0%) underwent total thyroidectomy. Among hemithyroidectomy patients, 174 (71.3%) proceeded to completion thyroidectomy. Among completion thyroidectomy patients, residual disease was found in 63 (36.2%). Univariate analysis showed no clinicopathological differences between those with and without residual disease. On multivariate analysis, male sex (OR 4.20, 95% CI [1.13, 15.63], $p = 0.034$) was independently associated with higher odds of residual disease in the completion lobe, while papillary subtype was associated with a lower odds of residual disease (OR 0.12, 95% CI [0.03, 0.50], $p = 0.0045$).

**Conclusions:** Larger nodule size and adverse pathological features were associated with higher rates of completion thyroidectomy. Among those undergoing completion surgery, male sex independently predicted residual disease, while papillary histology was independently associated with reduced risk.

**Open Access** This is an open-access article distributed under the terms of the Creative Commons Attribution 4.0 International License, which permits unrestricted use, distribution, reproduction, and adaptation in any medium, provided the original work is properly attributed.


Subjects: Artificial Intelligence in Surgery

38 Patient perceptions on artificial intelligence in surgery

Smyth N.M. 1 2
Narsiman A. 3 1
Canavan C.T. 4 1
Chandavarkar R. 1 5
Toncheva D. 1
Hill G. 1
Fleming L. 1
Ranatunga A. 1
Quirke M. 6
Cahir C. 7
Hill A.D.K. 1 5
Healy N. 8
1 Department of Surgery, Royal College of Surgeons Ireland, Dublin, Ireland
2 Department of Surgery, Conn olly Hospital, Blanchardstown, Ireland
3 Department of Surgery, Galway University Hospital, Galway, Ireland
4 Department of Surgery, Waterford University Hospital, Waterford, Ireland
5 Department of Surgery, Beaumont Hospital, Dublin, Ireland
6 Department of Emergency Medicine, Beaumont Hospital, Dublin, Ireland
7 Data Science Centre, Royal College of Surgeons in Ireland, Dublin, Ireland
8 Department of Radiology, Beaumont Hospital, Dublin, Ireland

131 AI driven wound assessment using image analysis

Shakir M.
Manchester NHS Foundation Trust, Manchester, UK

183 Bridging the gap in rehabilitation: real-time AI monitoring for home-based physiotherapy

Pattnaik S. 1
Pattnaik S. 2
1 Lewisham and Greenwich NHS Trust, London, UK
2 Manipal Institute of Technology, Udupi, India

197 CaudaNet - automated Cauda Equina compression identification on MRI scans

Biswas S. 1
Guo L. 2
George K.J. 1
1 Manchester Centre for Clinical Neurosciences, Manchester, UK
2 Manchester Metropolitan University, Manchester, UK

389 Prediction of day case discharge failure in total hip and knee arthroplasty using supervised machine learning in a non-selective enhanced recovery system

Selim A. 1
Ibrahim A. 1
Warren R. 2
Graham N. 2
Redfern D. 2
Thomas G. 2
1 University Hospitals of North midlands NHS trust, Stoke on Trent, UK
2 Keele university, Stoke on Trent, UK

Subjects: Early-Stage Innovation

53 Revolutionising anatomy education: the impact of virtual reality workshops on anatomy teaching

Ha J. 1
Chang F. 2
Mahmood A. 3
Yong C. 4
Salih A. 3
Ukpeh P.l 3
Abdalla L. 5
Gan A. 6
Chin K.Jian 5
Gullapalli S. 6
Gupta K. 7
Krishnan K. 7
Balan A. 8
Tannirandorn P. 4
Fernandez N.S. 9
Ali I. 3
Samanta A. 3
Kiew C.Y.K. 3
Lee J. 10
Fareed F.F.I 9
Teji M. 11
Gianchandani A. 3
Yasini A. 12
Khamise A. 9
Owens D. 13
Mortimer J. 14
Dhanda J. 15
1 University of Bristol, Bristol, UK
2 Cardiff University, Cardiff, UK
3 Imperial College London, London, UK
4 University College London, London, UK
5 Barts and the London, London, UK
6 University of Birmingham, Birmingham, UK
7 University of Leeds, Leeds, UK
8 University of Warwick, Warwick, UK
9 University of Buckingham, Buckingham, UK
10 King’s College London, London, UK
11 King's College London, London, UK
12 University of Central Lancashire, Preston, UK
13 University Hospital of Wales, Cardiff, UK
14 University of Edinburgh, Edinburgh, UK
15 Queen Victoria Hospital, East Grinstead, UK

113 Virtual reality teaching of the radial forearm flap: demonstrating the value of short, immersive sessions in overcrowded surgical curricula

Chu K.K.Y. 1
Chiara A. 1
Rehman U. 1
Sarwar M.S. 1
Dhanda J. 2
1 UK Plastics Research Collaborative (UKPRC), London, UK
2 Virtual Reality in Medicine and Surgery (VRiMS), London, UK

282 Escape the Operating Room: A Gamified Experience for Surgical Decision-Making

Dandiganahalli Channappa J.
Vasant A.
Ashford and St Peter's Hospital, Chertsey, UK

320 AI-driven aphasia-friendly communication tool to enhance perioperative consent and patient understanding

Sahoo A.S. 1
Singh A. 2
1 University of Lancashire, Preston, UK
2 TensaX Innovation Lab, Jaipur, India

Subjects: Clinical Research

247 Multimodal analysis of surgical trainees: exploring correlations between traditional and modern assessment

Broadbent R. 1
Murray J. 1
Howie E. 2
Clarke R. 3
Totton N. 4
Peckham-Cooper A. 5
Church H. 6
Yule S. 7
Tomlinson J. 1
1 Sheffield Teaching Hospitals, Sheffield, UK
2 Surgical Sabermetrics Laboratory, Usher Institute, University of Edinburgh, Edinburgh, UK
3 NHS England, Yorkshire and Humber School of Surgery, Yorkshire, UK
4 Sheffield Centre for Health and Related Research, University of Sheffield, Sheffield, UK
5 Leeds Institute of Emergency General Surgery, St James’s University Hospital University of Leeds, Leeds, UK
6 Faculty of Medicine and Health Sciences, University of Nottingham, Nottingham, UK
7 Clinical Surgery, University of Edinburgh & Royal Infirmary of Edinburgh, Edinburgh Surgical Sabermetrics Group, University of Edinburgh, Edinburgh, UK

353 Predictive validity of the ACS NSQIP surgical risk calculator for postoperative complications and length of stay after lung volume reduction procedures: a single-institution study

Villavicencio Clayton S.G. 1 2
Alzouabi M. 3
Brozik J. 4
Oey I. 2
Dawson A.G. 2
Rathinam S. 2
Greening N. 5 6
Caruana E.J. 2 6
1 University of La Laguna Medical School, La Laguna, Spain
2 Thoracic Surgery, Glenfield Hospital, Leicester, UK
3 Medical School, University of Leicester, Leicester, UK
4 Radiology, Glenfield Hospital, Leicester, UK
5 Department of Respiratory Sciences, University of Leicester, Leicester, UK
6 Institute for Lung Health, NIHR Leicester BRC, Leicester, UK

359 Endoscopic flexor hallucis longus transfer with interference screw and additional tension slide cortical button for chronic achilles tendon rupture

Newton A.C. 1
Franklin S. 1
Lewis T.L. 1
Mehrotra S. 2
Vignaraja V. 1
Ray R. 1
1 King’s Foot and Ankle Unit, King’s College Hospital NHS Foundation Trust, UK, London, UK
2 University of Sheffield, Sheffield, UK

361 Preliminary radiographic classification of first metatarsal osteotomy healing following minimally invasive hallux valgus surgery

Lewis T.L. 1
Mehrotra S. 2
Kaplan J. 3
Gonzalez T. 4
Morales S. 5
Goff T.J. 6
Vignaraja V. 1
Newton A.C. 1
Ray R. 1
Lam P. 7
1 King’s Foot and Ankle Unit, King’s College NHS Foundation Trust, London, UK
2 Sheffield Medical School, University of Sheffield, Sheffield, UK., Sheffield, UK
3 Duke University Orthopedics, Durham, NC, USA., Durham, USA
4 School of Medicine, University of South Carolina, Columbia, SC, USA
5 Orthopedic Surgery Department, Pontificia Universidad Católica de Chile, Santiago, Chile
6 Mid Yorkshire Hospitals NHS Trust, Wakefield, UK
7 Orthopaedics and Arthritis Specialist Centre, Sydney, Australia

444 Long-term outcomes of anastomotic urethroplasty for PFUI in a resource-limited setting

Hussain Mushtaq 1 2
Naz Kanwal 2
Zulfiqar Mazahir 2
Abidi Syed Saeed 2
1 Queen Elizabeth Hospital, Birmingham, UK
2 Sindh Institute of Urology and Transplantation, Karachi, Pakistan

Subjects: QI and audit

271 Reconstruction versus below-knee amputation for complex foot trauma: a retrospective cohort study

Poon Katherine 1
Zargar Ahmad 1
Karia Chiraag 2
Taylor Martin 2
Harwood Paul 2
Bakhsheyesh Peyman 2
Fostee Patrick 2
Rollet Rebecca 2
Bhat Waseem 2
West Christopher 2
Leonard David A 2
1 University of Leeds, Leeds, UK
2 Leeds Teaching Hospitals NHS Trust, Leeds, UK

299 Impact of undergraduate surgical conferences on medical student engagement, surgical skills confidence and career aspirations: a two-year service evaluation project

Varma Nikhita 1
Camderman Dione 1
Chauhan Amir 1
Tan Jacob 2
Kiwan Omar 1
Wong Evelyn 3
Oskui Yasmin 4
Lord Mathilda 5
Tooke James 6
Lamb Sophia 1
Sharrock Martin 7
McBride Richard 8
1 Faculty of Biology, Medicine and Health, The University of Manchester, Manchester, UK
2 Mersey and West Lancashire Teaching Hospitals NHS Trust, Liverpool, UK
3 Nottingham University Hospitals NHS Trust, Nottingham, UK
4 Wrightington, Wigan and Leigh Teaching Hospitals NHS Foundation Trust, Manchester, UK
5 Chelsea and Westminster Hospital NHS Foundation Trust, London, UK
6 Stockport NHS Foundation Trust, Manchester, UK
7 Department of Orthopaedic Surgery, Sheffield University Teaching Hospitals Foundation Trust, Sheffield, UK
8 Department of Vascular Surgery, Lancashire Teaching Hospitals NHS Foundation Trust, Preston, UK

302 Are out-of-hours transfers for MRI in suspected cauda equina syndrome justified?

Lester Mohammed 1
Covington Sophie 2
Abdi Zakee 3
Jalal Arif 4
Toma Ahmed K. 1
Sayal Parag 1
Prezerakos Georgios 1
Pandit Anand S. 1
1 Victor Horsley Department of Neurosurgery, National Hospital for Neurology and Neurosurgery, London, UK
2 Division of Medicine, University College London, London, UK
3 Barts and the London School of Medicine and Dentistry, Queen Mary University of London, London, UK
4 Imperial College Healthcare NHS Trust, London, UK

311 Cutting costs and boosting efficiency: reducing routine group & save testing in flexible ureterorenoscopy for better resource use and patient care

Shadananan Vishnu
Dhariwal Randeep
Arumuham Vimoshan
University College London Hospitals NHS Foundation Trust, London, UK

429 When the bone flap hits the floor: developing the first SOP for dropped bone flaps in neurosurgery at Oxford

Sivanathan Shayndhan
Boukas Alexandros
Oxford University Hospitals, Oxford, UK

443 Improving inpatient administration of intravenous zoledronic acid following hip fracture: a closed-loop audit.

Wilson Zarah
Rasool Muhammad Umer
Ghazi Muhammad Anas
Naureen Mahwish
Manchester University NHS Foundation Trust, Manchester, UK

Subjects: Collaborative Research

26 Neurosurgeons’ leadership styles and their association with team performance and patient outcomes: a national multi-modal analysis

Saghebdoust Sajjad 1 2
Jouzdani Ali Fathi 2
Zahmatkesh Mohammad Reza Rouhbakhsh 2
Soltani Ghasem 3
Zare Reza 2
1 Department of Neurosurgery, Queen's Hospital, Barking Havering and Redbridge University Hospitals NHS Trust, Romford, UK
2 Department of Neurosurgery, Razavi Hospital, Mashhad, Iran, Islamic Republic of
3 Department of Anesthesiology, Razavi Hospital, Mashhad, Iran, Islamic Republic of

339 PECHA study: percutaneous cholecystostomy in acute cholecystitis, a multicentre cohort study

Karagiannidis Georgios 1
Uka Dardan 2
Iacob Maria 3
Labinoti Roland 3
Salem Yousef 2
Ergisi Mehmet Mehmet 4
Hall Sophie 2
Sinclair Martin 2
1 Imperial College London, London, UK
2 ESNEFT, Ipswich, UK
3 Ipswich Hospital NHS Trust, Ipswich, UK
4 NNUH, Norwich, UK

342 Influence of referral pathways and consultant subspecialty on morbidity and mortality in acute severe pancreatitis: a multicentre observational study

Karagiannidis Georgios 1
Lone Ahmed 2
Rahman Zahran 2
Pathak Samir 3
Sinclair Martin 2
1 Imperial College London, London, UK
2 ESNEFT, Ipswich, UK
3 Leeds Teaching Hospitals, Leeds, UK

351 ETHOS study: evaluation of hernia treatment and outcomes in pregnancy, a multicentre, cohort study

Karagiannidis Georgios 1
Banham Evie 2
Zaman Sadia 3
Tee Jie Ying 4
Gupta Ankit 5
Barry Charles 3
Salim Shabman 6
Hakim Rebecca 6
Hunter Rachel 7
Sinclair Martin 4
1 Imperial College London, London, UK
2 Ipswich Hospital NHS Trust, Ipswich, UK
3 Nottingham NHS trust, Nottingham, UK
4 ESNEFT, Ipswich, UK
5 Leeds Teaching Hospitals, Leeds, UK
6 Bristol Royal Infirmary, Bristol, UK
7 Airedale NHS Trust, Bradford, UK

Subjects: Robotics and Digital Surgery

258 Introducing robotic inguinal hernia repair: early outcomes from a UK NHS trust

Bartlett Robyn
Kent Olivia
Cowie Stuart
Horgan Liam
Northumbria Healthcare NHS Foundation Trust, North Shields, UK

277 Virtually seamless: virtual reality for colorectal anastomosis training

Mahmoud Salma 1
Elcock Katherine 2
Harji Deena 2
Bowers Michael 3
Orsucci Sonia 3
1 Mersey and West Lancashire Teaching Hospitals, Liverpool, UK
2 Manchester Foundation Trust, Manchester, UK
3 Johnson and Johnson MedTech, Manchester, UK

294 Survey of robotic thoracic surgery training in the UK and Ireland: interim analysis of a national, multi-centre survey

Jesani Hannah 1
Hundle Aaron 2
Patel Akshay 2
Gooseman Michael 3
1 University Hospitals Coventry & Warwickshire, Coventry, UK
2 UHB NHS Foundation Trust, Birmingham, UK
3 Hull University Teaching Hospitals NHS Trust, Hull, UK

307 Minimally Invasive but Donor-Centric: A Systematic Review and Meta-Analysis of Robotic Versus Open Living-Donor Hepatectomy

Sriram Vibha
University of Buckingham, Buckingham, UK

313 A systematic review and meta analysis comparing clinical and functional outcomes of Mako-assisted versus conventional total knee replacement (PROSPERO ID: CRD420251115864)

Sahoo Aman Saswat 1
Tiku Arjun 1
Chandanani Vishal 2
Shienh Preet Noor 1
Thakur Abdul Wadood Iqbal 3
1 University of Lancashire, Preston, UK
2 University Hospitals Bristol and Weston NHS Foundation Trust, Bristol, UK
3 Betsi Cadwaladr University Health Board, Tremadog, UK

325 Colorectal trainees as primary console surgeons in elective robotic assisted surgery: evaluating outcomes of a robotic colorectal training programme

Akbar Muhammad Bilal
Aung Aye
Abduldaiem Yousef
Maghazi Maaz
Carden Claire
Shah Adarsh
NHS Tayside, Dundee, UK

Subjects: Systematic Review and Meta-Analyses

191 Comparing post-operative wound outcomes of negative pressure wound therapy (NPWT) vs standard dressings in emergency laparotomies: a systematic review and meta-analysis

Li Sze Wai Rosa 1
Kritika FNU 2
Koziol Mateusz 3
1 Cardiff University, Cardiff, UK
2 Department of General Surgery, Indira Gandhi Institute of Medical Sciences, Patna, India
3 Jagiellonian University Medical College, Krakow, Poland

201 The risk of recurrent anterior cruciate ligament injury in male and female football players following anterior cruciate ligament reconstruction: a systematic review and meta-analysis

Wood Henry 1
Byrne Chris 2
1 University of Exeter Medical School, Exeter, UK
2 Public Health and Sport Sciences, University of Exeter, Exeter, UK

336 Autologous lymphatic vessel transplantation for extremity lymphedema: a systematic review and meta-analysis

Wong Zhen Yu 1
Lai Luanne 2
Faderani Ryan 3
Kanapathy Muholan 3
Mosahebi Afshin 3
1 Welsh Centre for Burns & Plastic Surgery, Morriston Hospital, Swansea, UK
2 Queens Medical Centre, Nottingham, UK
3 UCL, London, UK

414 Focused appendiceal CT as an innovative diagnostic pathway, a systematic review and meta-analysis

Shurovi Badrun 1
Samra Benjamin 2
1 Royal London Hospital, London, UK
2 University Hospitals Birmingham, Birmingham, UK

434 Surgical versus nonsurgical management of distal radius fractures: a systematic review and meta-analysis highlighting functional outcomes and age-based considerations

Ghazi Muhammad Anas
Rasool Muhammad Umer
Wilson Zarah
Manchester University NHS Foundation Trust, Manchester, UK

Subjects: Global Surgery

23 Improving the timeliness of surgical antibiotic prophylaxis in cranioplasty: a three-cycle closed-loop audit at a tertiary neurosurgical center

Saghebdoust Sajjad 1 2 3
Vasilica Anca-Mihaela 4
Jouzdani Ali Fathi 3
Zare Reza 3
Mehrizi Mohammad Ali Abouei 2
1 Department of Neurosurgery, Queen's Hospital, Barking Havering and Redbridge University Hospitals NHS Trust, Romford, UK
2 Department of Neurosurgery, Shahid Kamyab Hospital, Mashhad University of Medical Sciences, Mashhad, Iran, Islamic Republic of
3 Department of Neurosurgery, Razavi Hospital, Mashhad, Iran, Islamic Republic of
4 University College London (UCL), Medical School, London, UK

135 Laparoscopic versus open cholecystectomy in low- and middle-income countries: a systematic review and meta-analysis of perioperative and postoperative outcomes

Abdelnour David Safwat Thabet 1
Ahmed Ali Yasen Mohamed 1
Mohammed Safeya 2
Louw Johnelize 3
Chao Tiffany E 3
Chu Kathryn 3
1 Kettering general Hospital, Kettering Northamptonshire, UK
2 Ibrahim Malik Teaching Hospital, Khartoum, Sudan
3 Stellenbosch University, Stellenbosch, South Africa

198 Brachial plexus injuries in Cambodia: demographics, interventions, and post-operative outcomes

Wood Henry 1
Jowett Vivien 2
Tan Vichet 3
Kim Yong-June 2
1 Royal Cornwall Hospital, Truro, UK
2 Children's Surgical Centre, Phnom Penh, Cambodia
3 Children's Surgical Centre, Phnom Penh, Cambodia

200 Outcomes of collagen and conventional dressings in diabetic foot ulcers: a comparative study in Bangladesh

Rahman Md Arifur
Kettering General Hospital NHS Trust, Kettering, UK

308 Breaking barriers: establishing robotic surgery in a developing country

Hussain Mushtaq 1 2
Mohsin Rehan 2
Qureshi Harris 2
Leghari Riaz 2
Mahar Naveed 2
1 Queen Elizabeth Hospital, Birmingham, UK
2 Sindh Institute of Urology and Transplantation, Karachi, Pakistan

355 Systematic review of nonoperative joint-preserving strategies in sickle cell disease-related avascular necrosis: implications for low-resource healthcare systems

Oladeji Emmanuel 1 2
Olayode Oluwatobi 1 3
Zubair Abdulahi 1
Okonkwo Patrick 1
Omerenma Onyeka 1
Obakponovwe Oghofori 2
1 Surgery Interest Group of Africa, Lagos, Nigeria
2 St Richard's Hospital, Chichester, UK
3 Obafemi Awolowo University Teaching Hospital, Ile-Ife, Nigeria

427 Low level of awareness regarding prostate cancer and screening among men at risk in rural Gezira State, Central Sudan: a multi-centered cross-sectional study

Alrahman Hamid Ali Mohamed Abd
Elamin Dafalla
Faculty of Medicine, University of Gezira, Wad Madani, Sudan

Subjects: Best Top 5

184 Efficient and explainable machine learning for dermoscopic image classification

Pattnaik Saphalya 1
Pattnaik Sanman 2
1 Lewisham and Greenwich NHS Trust, London, UK
2 Manipal Institute of Technology, Udupi, India

281 Extent of resection in meningioma surgery: concordance and prognostic value of surgical, radiological, and volumetric assessments

Deehan Matthew 1
Mallon Dermot 2
Mathew Shalwin 1
Pace Gillian 1
Kitchen Neil 1
Hyare Harpreet 2
Nachev Parashkev 3
Marcus Hani 1 4
Pandit Anand 1
1 Victor Horsley Department of Neurosurgery, National Hospital for Neurology and Neurosurgery, London, UK
2 Lysholm Department of Neuroradiology, National Hospital for Neurology and Neurosurgery, London, UK
3 High Dimensional Neurology, Queen Square Institute of Neurology, London, UK
4 TeQ Group, Queen Square Institute of Neurology, London, UK

285 NHS oral cancer patient-facing information: enhancing readability and preserving content with large language models

Baczynska Agata 1
Rehman Umar 2
Gohari Shireen 3
Chu Kelly 4
Sarwar Mohammad 5
Brennan Peter 6
1 Imperial College London, London, UK
2 UCL Division of Surgery and Interventional Science, London, UK
3 St Georges University Hospital - NHS trust, London, UK
4 Department of Plastic Surgery, Lister Hospital, Stevenage, UK
5 Department of Oral and Maxillofacial Surgery, Queen Victoria Hospital NHS Foundation Trust, East Grinstead, UK
6 Department of Oral and Maxillofacial Surgery, Queen Victoria Hospital NHS Foundation Trust, Portsmouth, UK

447 Total thyroidectomy or hemithyroidectomy for low-risk differentiated thyroid carcinoma?

McGarry Jennifer 1
Zaborowski Alexandra 1
McShane Nicola 1
Alkeni Badriya 2
Walton Tessa 2
Kearns Emma 3
Choo Ryan Rui Jye 3
McInerney Niall 3
Crowley Rachel 4
Bell Maricia 5
Moran Tom 6 3
O'Duffy Fergal 6 3
Young Orla 7
Evoy Denis 1
Lowery Aoife 2
Kerin Michael 2
Prichard Ruth S 1
1 Department of Breast and Endocrine Surgery, St Vincent’s University Hospital, Dublin, Ireland
2 Department of Breast and Endocrine Surgery, Galway University Hospital, Galway, Ireland
3 Department of Otorhinolaryngology/Head and Neck Surgery, Mater Misericordiae University Hospital, Dublin, Ireland
4 Department of Endocrinology, St Vincent’s University Hospital, Dublin, Ireland
5 Department of Endocrinology, Galway University Hospital, Galway, Ireland
6 Department of Otorhinolaryngology/Head and Neck Surgery, St Vincent’s University, Dublin, Ireland
7 Department of Otorhinolaryngology/Head and Neck Surgery, Galway University Hospital, Galway, Ireland

